# Geographic and Battery-Aware Kubernetes Scheduling for IoT Sensing

**DOI:** 10.3390/s26217003

**Published:** 2026-11-05

**Authors:** George Koufoudakis

**Affiliations:** Department of Informatics, Ionian University, 49100 Corfu, Greece; gkoufoud@ionio.gr

**Keywords:** Kubernetes, Internet of Things, edge computing, container scheduling, geographic scheduling, battery state, measurement placement

## Abstract

In Internet of Things (IoT) applications, measurements may be requested at specified points and assigned to worker nodes whose locations and reported battery states differ. For such workloads, GeoBattery was implemented as an optional Kubernetes Scheduling Framework plugin. Target coordinates are read from pod annotations, while fresh ordinal battery reports are obtained from node conditions. Eligible nodes are then ranked by a configurable combination of Euclidean distance and reported battery state, following the standard feasibility checks. The plugin was evaluated against generated workloads in a Go simulator without a running cluster. Proximity, battery, and combined policies were compared over 1000 timesteps per run. A default profile was examined separately, and sensitivity was studied by changing one factor at a time. Under combined scoring, intermediate distance and completion outcomes were obtained relative to the policies using either input alone, with small differences in modeled lifetime during observation. These findings are confined to the generated workloads and stated battery assumptions. Physical energy use, battery endurance, and sensing quality remain to be measured on devices, and scheduler behavior requires evaluation in a live cluster.

## 1. Introduction

In sensing applications, jobs of short duration may be assigned to edge nodes for measurements at specified points. A worker is selected for each pod through the Kubernetes Scheduling Framework, where feasibility filtering is followed by node scoring [1]. The use of Kubernetes for edge and IoT workloads has been reviewed by Senjab et al. [2]. For battery powered nodes, the placement decision may also be affected by the reported reserve and the distance from the requested point.

Earlier Kubernetes schedulers have addressed energy use and interference [3], network conditions [4], contextual placement [5], delay [6], and topology and service level objectives [7]. Greener placement [8] and policies that adapt to runtime conditions in fog and IoT workloads [9] have also been studied. The inputs and evaluation settings differ across these approaches. A battery report describes a node’s current reserve. Energy consumption and battery endurance require separate measurements. Geographic distance describes spatial separation, whereas network latency depends on the communication path. Sensing accuracy is treated as a separate application outcome.

GeoBattery was implemented as an optional Kubernetes Scheduling Framework plugin for sensing tasks with specified target points. Each measurement pod is supplied with a target in a shared Cartesian frame, and node coordinates are accompanied by a fresh ordinal battery condition. The feasible set is established through the standard Kubernetes filters and GeoBattery validation. The retained nodes are ranked using configurable battery and distance weights. Ordinary pods continue to be handled by the default profile when the default scheduler name is used.

The effects of target distance and fresh ordinal battery reports on node ranking, placement distance, request completion, and modeled depletion were examined with generated workloads. Kubernetes 1.34.1 Scheduling Framework plugins were executed directly against generated clusters under synthetic battery assumptions, without a running cluster. Each policy run in the matched comparison covered 1000 timesteps. Intermediate mean distance and request completion were obtained when both inputs were used, while restricted mean lifetime changed little during observation. The default profile was evaluated separately because a different set of eligible nodes was used. Drain, initial battery, weights, and distance scale were varied in the tested scenarios, with their effects recorded for placement and completion. Distance was interpreted as spatial separation and battery outcomes as depletion under the model. Selection latency, total simulator CPU time, and peak process memory were measured in a separate sweep of generated clusters with 200–2000 nodes.

The contribution consists of the plugin input contract, its configurable scoring policy, and an evaluation of placement decisions, request outcomes, modeled depletion, and simulator cost. Internal policies were compared under matched conditions by executing the scheduling plugins within the simulator process. The default profile is reported separately, since subarea affinity changes the eligible nodes. The resulting evidence is limited to the generated workloads, battery assumptions, score settings, and evaluation method. Earlier studies are used for context, while direct comparisons of practical performance require a shared workload and implementation. Device measurements and live cluster tests are needed for physical energy use, sensing outcomes, and scheduler behavior in deployment. Related work is reviewed in Section 2. The system model and problem are defined in Section 3, followed by the plugin implementation in Section 4. Evaluation results are presented in Section 5 and discussed in Section 6, with conclusions and further work given in Section 7.

## 2. Related Work

Several scheduling objectives have been studied for edge and IoT environments. Energy use and interference are modeled in KEIDS for Industrial IoT placement [3], while network information is used by Santos et al. for fog resource provisioning [4]. Physical, operational, and network context were incorporated into 5G edge scheduling by Ogbuachi et al. [5]. Processing and network delay are addressed in DACS [6], and topology and service level objectives are considered in Polaris across cloud, edge, and IoT clusters [7]. Runtime telemetry is used in Chronos to adapt scheduling policies [9]. In CosmoSpan, workload descriptions guide placement across federated clusters [10], whereas computational and network energy are considered in CODECO [8]. The reported results are discussed within each study’s settings, since their inputs, workloads, platforms, baselines, and measures differ.

### 2.1. Energy, Battery, and Greenness Objectives

In KEIDS, Industrial IoT container placement is formulated as a multiobjective optimization of energy use, carbon footprint, and application interference, subject to resource, deadline, and replication constraints [3]. Google cluster traces, four assumed geographically distributed clusters, and a MOSEK optimization model are used in its evaluation. FCFS and KEIDS variants with individual objectives are used as comparators [3]. Improvements over FCFS are reported for the study’s energy utilization, carbon footprint, and interference measures [3].

Battery state is used directly in SOCCS, the Kubernetes scheduler proposed by Llorens-Carrodeguas et al. for battery-powered edge systems [11]. State of charge is estimated from current measurements, and the effect of assigning an event is predicted by regression. Predicted charge and CPU utilization are combined in node scoring, with battery thresholds and event deadlines also considered. Event workloads were evaluated on four Raspberry Pi nodes with batteries and power meters, using native Kubernetes and least-loaded scheduling as comparators.

In CODECO, placement across the continuum from IoT devices to edge and cloud systems is informed by computational energy monitoring and network energy indicators [8]. Tests on ARM-based far-edge devices are reported in the first arXiv version, where computational and network energy are treated as separate scheduling dimensions [8].

A custom K3s scheduler was described in [12] and evaluated on three Raspberry Pi nodes. CPU and memory availability, bandwidth, and an energy-related metric were used in node selection. The energy metric was derived in software, and direct power instrumentation was identified as a next validation step.

Chronos adapts scheduling policies for fog and IoT workloads using runtime telemetry and performance data collected over time [9]. It was evaluated on a testbed of single-board computers with workloads that stressed the network, disk, and CPU. Default Kubernetes scheduling and random scheduling were used as baselines [9]. Relative to the default scheduler, reductions of up to 23% in network latency, 42% in disk write time, and 20% in response time for CPU-intensive workloads are reported [9]. These are service measurements for the workloads used on that testbed.

### 2.2. Delay, Network, and Context-Aware Placement

For delay-sensitive and data-intensive fog applications, including Smart City and environmental monitoring use cases, Santos et al. incorporate network infrastructure information into Kubernetes resource provisioning [4]. Lower network latency than with default scheduling is reported in the evaluated scenarios, where network state is a central input to the decision [4].

For Industrial IoT applications at the 5G edge, physical, operational, and network conditions are considered in the context-aware Kubernetes scheduler developed by Ogbuachi et al. [5]. Wi Fi and cellular configurations, including 4G and non-standalone 5G, were evaluated as communication and infrastructure contexts [5]. Toka addresses reliable, low-latency edge computing by provisioning resources for distributed edge nodes and latency-sensitive applications [13].

DACS first filters heterogeneous IoT edge nodes by available CPU, memory, and network resources, then ranks the remaining nodes by estimated processing and network delays [6]. The evaluation was conducted on a VMware-based Kubernetes cluster with one master and three workers. Two workload scenarios and low and high load conditions were included. The kube-scheduler, ElasticFog, TSRA, NAS, and KCSS were used as baselines [6]. Shorter total completion times are reported for DACS in the tested scenarios, although the differences vary with load, setting, and comparator [6].

### 2.3. Locality, SLOs, and Extensible Scheduling

Polaris is a framework for scheduling across cloud, edge, and IoT clusters with attention to edge location and service level objectives. Physical topology, network quality of service, locality, and scheduler extension points are incorporated [7]. Geographic and topological placement are therefore treated as relevant concerns in edge scheduling [7].

Bregar and Jurić describe CosmoSpan, a platform for multiple edge and cloud clusters where workload descriptions and collected telemetry guide placement across clusters and nodes [10]. CPU, memory, node uptime, network bandwidth, latency between workloads, fault tolerance, and unscheduled instances are among the placement criteria [10]. In synthetic KWOK evaluations, average Edge-Cloud Continuum Placement Scores are reported to be 41% above the default Kubernetes scheduler and 27% above Diktyo. An end to end scenario across three physical clusters was also used to assess how well the simulation results represented the system [10].

Application profiles are used by Mekki et al. to plan deployment and migration across cloud and edge infrastructures over time [14]. Cost and carbon footprint are combined subject to resource, bandwidth, user latency, availability, and migration constraints. Geographic location supplies regional carbon intensity, while locality to users is represented through latency requirements. In the manuscript, heuristic planning is compared with Gurobi solutions in synthetic scenarios, and migration downtime is examined separately with Kubernetes and K3s.

Policies concerned with energy, performance, and resource use are classified in the Kubernetes scheduling survey [2]. An extensible framework for SLO-aware scheduling is described through Polaris [7], while configurable policies based on telemetry are provided by Chronos. A distinction is made here between scheduler integration, through which a platform capability is provided, and the policy through which the application objective is expressed.

For serverless workloads, Angelelli et al. formulate two-level batch allocation across heterogeneous edge and cloud clusters, trading data transfer cost against makespan [15]. Linear programming and matching are followed by local scheduling that accounts for reusable container layers. Function profiles were measured using OpenWhisk on Kubernetes, while policy comparisons were simulated with Batsim and SimGrid against an ImageLocality-inspired baseline. FOA-Energy extends this formulation with energy profiles for functions and their container environments [16]. Physical profiling supplies its energy inputs, but the placement policies are compared in simulation with known batches and platform capacities. The two studies concern successive formulations in the same research line.

### 2.4. Adjacent Kubernetes Research

For machine learning inference, accelerator metrics and workload behavior are used to guide colocation and resource use in Kubernetes scheduling that accounts for GPUs [17]. FLEDGE provides Kubernetes-compatible orchestration for edge devices with limited resources through Virtual Kubelets. Its resource footprint was evaluated on Raspberry Pi hardware [18]. These studies address accelerator management and the cost of edge orchestration.

GAIKube presents a proactive orchestration framework for heterogeneous edge computing that uses generative AI to predict workload and resource requirements for placement decisions [19]. This predictive approach is intended for workloads with changing demand.

Prophet and long short-term memory models are used in autoscaling based on forecasts of HTTP request demand and estimated pod requirements [20]. Replica counts are controlled by autoscaling, while individual pods are assigned to nodes by the Kubernetes scheduler. The mechanisms therefore operate at different control points. Security misconfigurations in Kubernetes manifests were examined in an empirical study that presents SLI-KUBE [21]. These related studies address accelerators, edge orchestration, replica scaling, and deployment security.

Energy consumption, communication and service performance, topology, federated placement, and resource management are addressed in the reviewed studies. A narrower placement decision is considered in GeoBattery, where feasible nodes within a cluster are ranked by distance from each pod’s target point and a fresh ordinal battery report. Battery reserve is used as a placement preference. Target distance represents spatial separation, while energy consumption, network delay, and sensing accuracy are treated as separate outcomes. Policy conformance was checked with unit tests, and placement distance, request completion, and modeled depletion were examined with generated workloads. Experimental comparisons are confined to internal score variants and a separately reported default profile. The reviewed schedulers are used for literature context, with their results interpreted according to each study’s objectives, workloads, baselines, and metrics.

## 3. System Definitions and Problem Formulation

The placement decision is defined for one pod in a Kubernetes cluster whose worker nodes represent container-capable IoT edge devices. Kubernetes assigns pods to feasible nodes. Edge frameworks can add locality and service objectives [7]. Each request and worker node is represented by Cartesian *x* and *y* coordinates in meters within a shared local frame. Each node also reports a fresh ordinal battery state. Within the bounded study area, planar Euclidean distance is assumed to meet the placement requirements. Standard Kubernetes filters establish feasibility. GeoBattery then validates the coordinates and battery inputs before ranking the remaining nodes.

### 3.1. Cluster, Request, and Telemetry Model

Let N={1,…,n} denote the worker nodes visible to the scheduler, and let *p* denote a measurement pod. A fixed regional origin $x_{0}$ is chosen, and a two-dimensional Cartesian frame is defined with the *x*-axis pointing east and the *y*-axis pointing north. Coordinates are measured in meters. The requested target point is carried by the pod:

(1) $$x_{p}=\left(x_{p},y_{p}\right),$$

where $x_{p}$ and $y_{p}$ are the target’s east and north offsets. Node *i* is located at $x_{i}=\left(x_{i},y_{i}\right)$ and reports an ordinal battery band $q_{i}\in \left\{1,\ldots ,10\right\}$. In the simulator, a percentage $B_{i}\in [0,100]$ is assigned to band 1 for $0\leq B_{i}\leq 10$, band 2 for $10<B_{i}\leq 20$, and band 10 for $90<B_{i}\leq 100$. The order of these bands is preserved. Device-specific calibration is needed before equal band increments can be interpreted as equal changes in energy. All nodes and target points use the same local coordinate frame. The deployment area is assumed to be small enough for planar Euclidean distance to meet the required spatial accuracy. The region and coordinate frame are specified so that generated node locations and target points can be reproduced.

Finite *x* and *y* values in meters are supplied through the pod annotations listed in Table 1 and the corresponding node labels. A common origin and axis orientation are used for all positions. Euclidean distance is calculated by the scheduler, with negative node label values encoded using a leading m. Signed decimal values are used in pod annotations.

The Cartesian inputs $x_{p},y_{p},x_{i},y_{i}$ are finite real values in meters. Battery reports use the recognized BATTERYLEVEL1 through BATTERYLEVEL10 condition types. Exactly one condition must be True, and every other condition that is present must be False. Let *t* denote scheduler time at the start of a scheduling cycle, $t_{i}^{\mathrm{hb}}$ the heartbeat of the active battery condition, and τ the permitted report age. A report is considered fresh when

(2) $$0\leq t-t_{i}^{\mathrm{hb}}\leq \tau ,$$

and the active heartbeat is present. The battery input is therefore an ordinal category qualified by time. Continuous state of charge and power are separate measurements.

The request, node, and policy inputs are summarized in Table 2.

### 3.2. Cartesian Distance Model

For each node *i*, coordinate differences are defined as $\Delta x_{i}=x_{i}-x_{p}$ and $\Delta y_{i}=y_{i}-y_{p}$. The planar distance from the requested target to node *i* is calculated with the Euclidean norm

(3) $$d_{i}={∥x_{i}-x_{p}∥}_{2}=\sqrt{{\left(x_{i}-x_{p}\right)}^{2}+{\left(y_{i}-y_{p}\right)}^{2}},\;d_{i}\geq 0,$$

where $d_{i}$ is measured in meters. Under the bounded area assumption, this calculation is applied throughout the deployment frame.

**Figure 1:**
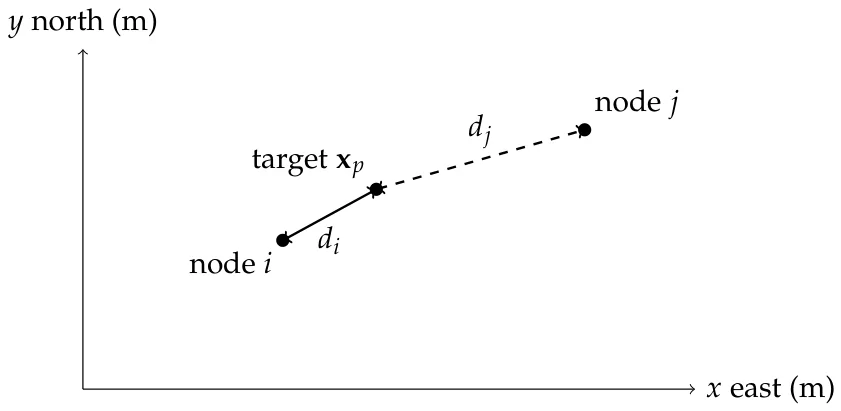
Local Cartesian representation of the target point and two worker nodes. The solid line shows the distance from the target to node *i*, and the dashed line shows the distance from the target to node *j*. Sensing-coverage regions and maximum-range boundaries are separate application constraints.

Figure 1 shows the Cartesian distance model, with east and north offsets in meters. Sensor coverage and application range thresholds are handled separately. Once feasibility and telemetry checks have been passed, a node remains a candidate regardless of its distance.

### 3.3. Feasibility and Candidate Set

Let $F_{K}\left(p\right)\subseteq N$ contain the nodes that pass the standard Kubernetes scheduling filters for pod *p*, including resource availability, applicable affinity, taints and tolerations, and other configured constraints [2]. Let $V_{i}$ indicate whether node *i* has valid coordinates and a valid, fresh battery report under the telemetry contract. The GeoBattery candidate set is

(4) $$C\left(p\right)=\{i\in F_{K}\left(p\right):V_{i}=\mathrm{true}\}.$$

Nodes with invalid coordinates or battery conditions are rejected during validation. Those that pass remain eligible at any distance. Distance affects only their proximity contribution. The application may impose sensor range and coverage constraints. Locality is a recognized concern in edge scheduling. Here, it is represented by a point distance preference applied after feasibility filtering [7].

### 3.4. Battery and Proximity Score

For each candidate i∈C(p), the normalized battery preference is

(5) $$b_{i}=\frac{q_{i}-1}{9},\;b_{i}\in [0,1],$$

where battery bands 1 and 10 correspond to the lower and upper endpoints of the normalized scale. The transformation preserves the order of the ten reported bands and assigns equal increments to adjacent steps. Each band remains an ordinal battery category. Continuous state of charge and energy consumption are separate quantities.

Let D>0 be the proximity scale in meters. The normalized proximity preference is

(6) $$p_{i}=\mathrm{clip}\left(1-\frac{d_{i}}{D},0,1\right),$$

where clip(z,0,1)=max(0,min(1,z)). At zero distance, $p_{i}=1$. It decreases linearly for $0<d_{i}<D$ and is zero for $d_{i}\geq D$. Feasibility is retained for candidates at or beyond *D*, which continue to contribute through their battery score.

Let $w_{b},w_{p}\geq 0$ be the battery and proximity weights, subject to $w_{b}+w_{p}>0$. The integer score returned for node *i* is

(7) $$S_{i}=\mathrm{round}\left[100\left(\frac{w_{b}}{w_{b}+w_{p}}b_{i}+\frac{w_{p}}{w_{b}+w_{p}}p_{i}\right)\right],\;S_{i}\in \left\{0,\ldots ,100\right\}.$$

The default settings are $w_{b}=w_{p}=1$, $D=2000/\sqrt{2}\;m$, and maximum battery age τ=300s. These values were used in the evaluation. Values for a deployment can be selected according to its objective and calibrated with measurements from physical devices. Configuration validation permits a maximum battery age of up to 86,400 s, although 300 s was used in the experiments. In the sensitivity campaign, the weight ratio and distance scale were varied separately. Alternative mappings of battery bands and energy calibration remain to be examined. Equal increments on the normalized scale express ordinal preference only. Device measurements are needed to determine whether these increments correspond to equal changes in available energy. Configuration validation also requires finite, nonnegative weights with a positive finite sum, a positive finite distance scale, and a battery age from 1 to 86,400 s.

### 3.5. Optimization Objective and Interpretation

When C(p) is nonempty, the placement decision is the maximum-score set

(8) $$M\left(p\right)=\underset{i\in C(p)}{arg max}S_{i}.$$

A selected node belongs to M(p). Several candidates may maximize the score when equal integer scores are assigned. The score defines the preference order. Ties are resolved by the active kube-scheduler implementation. If C(p)=⌀, the pod remains pending until another scheduling attempt.

**Figure 2:**
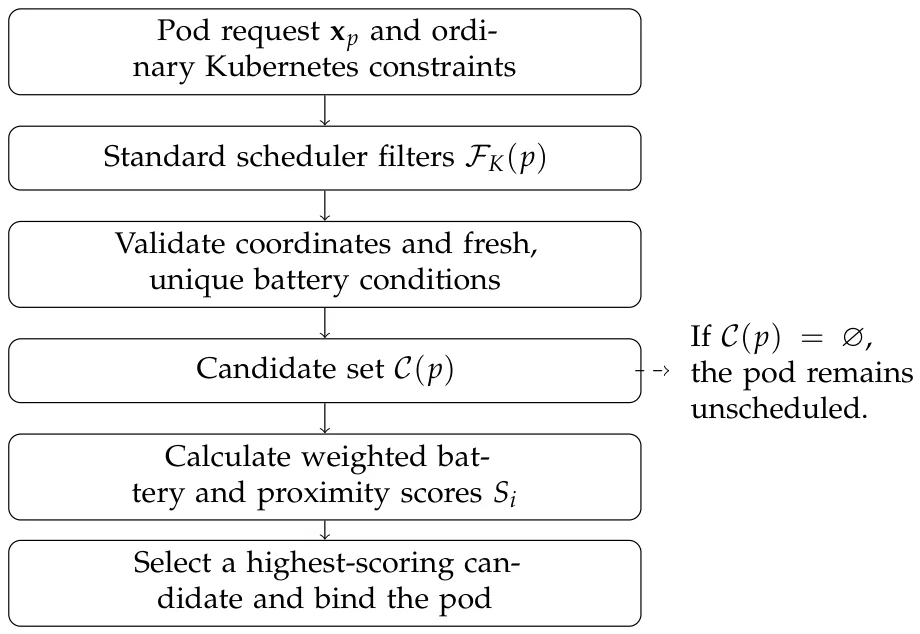
GeoBattery scheduling model from pod request to node selection. Solid arrows show the scheduling flow; the dashed arrow points to the note that the pod remains unscheduled if there are no candidates. The custom policy validates and scores candidates within the standard Kubernetes scheduling cycle.

The balance between reported battery state and point proximity is set through the weights. At equal battery bands, an equal or higher proximity score is assigned to the nearer node. At equal distances, the battery score is equal or higher for the node in the higher band. When both inputs differ, ranking is determined by their normalized weighted sum, so a farther node may be preferred if its battery contribution offsets the distance difference. The position of this ranking after feasibility filtering is shown in Figure 2.

### 3.6. Assumptions and Scope of the Model

Node coordinates and battery conditions are assumed to be supplied by an external telemetry process, with heartbeat timestamps comparable to the scheduler clock. The battery condition is reported on an ordinal scale with ten levels. Task energy demand and charge reservation are treated as separate resource management dimensions. One target point is supplied as geographic input, and distance is used as a scoring preference. Sensor reachability, measurement error, radio connectivity, network delay, energy consumption, and migration of pods that have already been bound each require their own model or measurements. Placement behavior is defined by Equations (3)–(8), through which candidate selection, score ordering, and point distance can be evaluated reproducibly.

## 4. GeoBattery Scheduler Design and Implementation

GeoBattery is implemented as a Kubernetes Scheduling Framework plugin and compiled into a custom kube-scheduler. Pods are handled by the dedicated profile when spec.schedulerName is set to iot-scheduler. Pods with the default scheduler name continue to be handled by the default profile. The policy is therefore applied only to measurement workloads that specify the custom scheduler, while the standard scheduling lifecycle is retained [2].

### 4.1. Plugin Architecture and Scheduling-Cycle State

The PreFilter, Filter, Score, and enqueue-extension interfaces are implemented by the plugin. During PreFilter, the pod’s Cartesian request point and the time at the start of the scheduling cycle are parsed and stored in cycle state. An unschedulable-and-unresolvable status is returned by PreFilter for a malformed or non-finite target.

**Figure 3:**
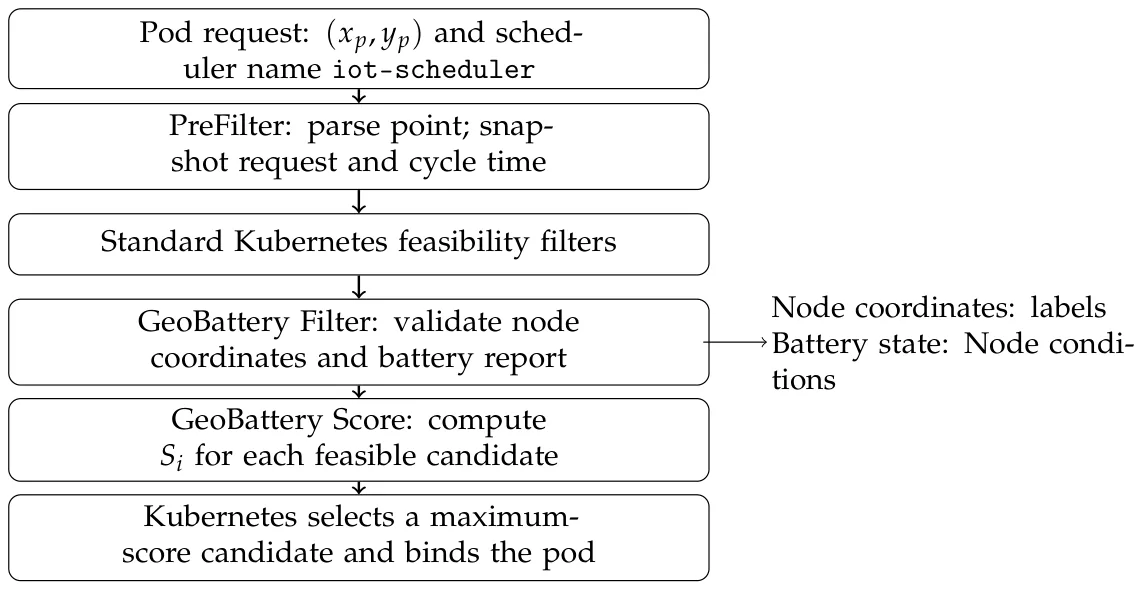
GeoBattery’s Scheduling Framework path. Standard filters and plugin validation establish the feasible set before score-based node selection.

The feasible set is established by the profile’s standard filters and GeoBattery’s Filter plugin. Node coordinates and battery conditions are checked, and an unschedulable-and-unresolvable status is returned for invalid locations or reports. Valid candidates are scored with the configured battery and proximity weights. A feasible node with the maximum score is then selected and bound through the standard Kubernetes operation, as shown in Figure 3.

### 4.2. Input Contract and Node Validation

Finite target coordinates in meters are supplied through the pod annotations iot.geobattery/x-m and iot.geobattery/y-m. Worker coordinates are supplied through the labels iot.example.org/x-m and iot.example.org/y-m, using the same origin and axis orientation. Negative node label values are encoded with a leading m. For example, m120 denotes −120 m, whereas signed decimal values are used directly in pod annotations.

Battery state is reported through Node conditions named BATTERYLEVEL1 through BATTERYLEVEL10. Under the convention used in the evaluation, band 1 represents 0–10%, band 2 represents more than 10% through 20%, and each following band spans the next ten percentage points. Band 10 represents more than 90% through 100%. This ordinal category is used by the scheduler, while continuous state of charge is supplied through a separate interface. Each recognized condition type may appear once. Exactly one level must be True, and every other present level must be False. A heartbeat timestamp within the configured age limit is required for the active condition, so that a unique ordinal battery value is current for the scheduling cycle. Coordinates and battery status are supplied by external reporters. Sensor acquisition and battery reporting remain separate deployment components.

### 4.3. Score Computation and Analytical Properties

For each candidate *i*, Section 3 defines the normalized battery preference $b_{i}=\left(q_{i}-1\right)/9$, clipped proximity preference $p_{i}=\mathrm{clip}\left(1-d_{i}/D,0,1\right)$, and rounded score $S_{i}$. The weights can be expressed in normalized form:

(9) $$\alpha =\frac{w_{b}}{w_{b}+w_{p}},\;\beta =\frac{w_{p}}{w_{b}+w_{p}},\;\alpha +\beta =1.$$

Before integer rounding, the continuous score is

(10) $${\tilde{S}}_{i}=100\left(\alpha b_{i}+\beta p_{i}\right),\;S_{i}=\mathrm{round}\left({\tilde{S}}_{i}\right).$$

For candidates satisfying $0<d_{i}<D$, the distance sensitivity of the continuous score is

(11) $$\frac{\partial {\tilde{S}}_{i}}{\partial d_{i}}=-\frac{100\beta }{D};\;\frac{\partial {\tilde{S}}_{i}}{\partial q_{i}}=\frac{100\alpha }{9}\;\mathrm{for}\;\mathrm{the}\;\mathrm{continuous}\;\mathrm{extension}\;\mathrm{of}\;\mathrm{the}\;\mathrm{ordinal}\;\mathrm{band}\;\mathrm{scale}.$$

The first derivative applies within the unsaturated proximity interval. For $d_{i}\geq D$, the proximity contribution is zero. The second derivative follows from the linear normalization used to order the ten discrete bands. Rounding can assign the same integer score to nearby continuous scores, producing ties.

For two candidates *i* and *j*, the continuous score of *j* exceeds that of *i* exactly when

(12) $$\alpha \left(b_{j}-b_{i}\right)+\beta \left(p_{j}-p_{i}\right)>0.$$

**Figure 4:**
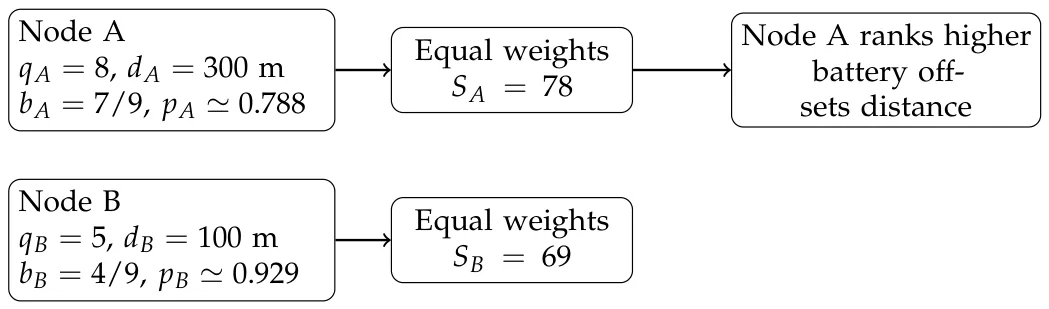
Illustrative score trade-off under equal battery and proximity weights. Node B is closer, while Node A’s higher battery band produces the larger rounded score. Values are calculated from the stated score model.

When battery and proximity preferences favor different nodes, their normalized weighted contributions determine the ranking. Figure 4 gives a worked example with equal default weights and $D=2000/\sqrt{2}$ m.

### 4.4. Scheduling Procedure and Profile Configuration

The scheduling cycle proceeds as follows:

1. The pod’s finite Cartesian point and one cycle timestamp are stored during PreFilter.
2. Nodes meeting resource, affinity, taint/toleration, coordinate, and battery-report requirements are retained by the standard Kubernetes filters and GeoBattery’s Filter plugin.
3. Each remaining candidate is evaluated by GeoBattery’s Score plugin using the normalized battery and Euclidean proximity preferences. An integer score in [0,100] is returned.
4. A highest-scoring feasible node is selected and bound through the standard operation. A pending pod can be reconsidered after registered Node or Pod events.

The deployed profile is named iot-scheduler. GeoBattery is enabled at PreFilter, Filter, and Score, with other Score plugins disabled and a weight of 1 assigned to GeoBattery. Battery weight, proximity weight, and maximum battery age are set through plugin arguments, while the validated default is used when a proximity scale is omitted. The scheduler’s own pod is assigned to the default scheduler, allowing the custom scheduler to start independently of its profile. Ordinary pods are also handled by the default scheduler. GeoBattery is used only by workloads that explicitly specify it.

### 4.5. Computational Cost and Operational Boundary

For a cluster of *n* nodes, at most *n* nodes are evaluated during Filter, after which the feasible candidates are scored. Two coordinates are parsed for each node, one Euclidean norm is calculated, and the battery condition list is inspected. The number of reported battery conditions is bounded. Policy work therefore grows linearly with the number of candidates, while score calculation requires constant work for each node. Only one request point and timestamp are stored in cycle state, so the amount of cycle data owned by the plugin remains constant.

The policy’s computational structure is described by this analysis, while runtime latency and scaling are measured separately because they also depend on the Kubernetes scheduler, cluster size, and node condition payloads. Each new pod is scheduled using the state observed during its scheduling cycle. Sensor and battery data are supplied by external reporters, and task energy reservation and migration of already bound pods are handled by separate lifecycle functions. The responsibilities of GeoBattery, the telemetry reporter, the measurement application, and the cluster are distinguished on this basis.

## 5. Evaluation and Results

The evaluation was carried out through a Go command-line tool without a running cluster. Kubernetes 1.34.1 Scheduling Framework plugins were executed in the same process against generated in-memory nodes, and request placements, distances, completion status, and run metadata were recorded. Four sets of results are presented. Weight-dependent decisions and placement across different subarea counts are examined first, followed by matched-policy comparisons, a separate default profile reference, and sensitivity tests over 1000 timesteps. Simulator cost is then examined as the node count changes. Thirty independent runs were completed for each condition, with matched seed streams used to retain the same initial layouts and request inputs within a comparison. Means and confidence intervals were calculated with the run as the independent unit, since requests and nodes were nested within runs. Battery depletion and lifetime are interpreted under the synthetic model. The protocol and supporting data are supplied in the Supplementary Material.

All experiments were run on an Apple M3 Pro laptop with 12 CPU cores and 36 GiB of installed memory. Further evaluation details are provided in the Supplementary Material.

### 5.1. Weight-Dependent Scheduler Decisions

**Figure 5:**
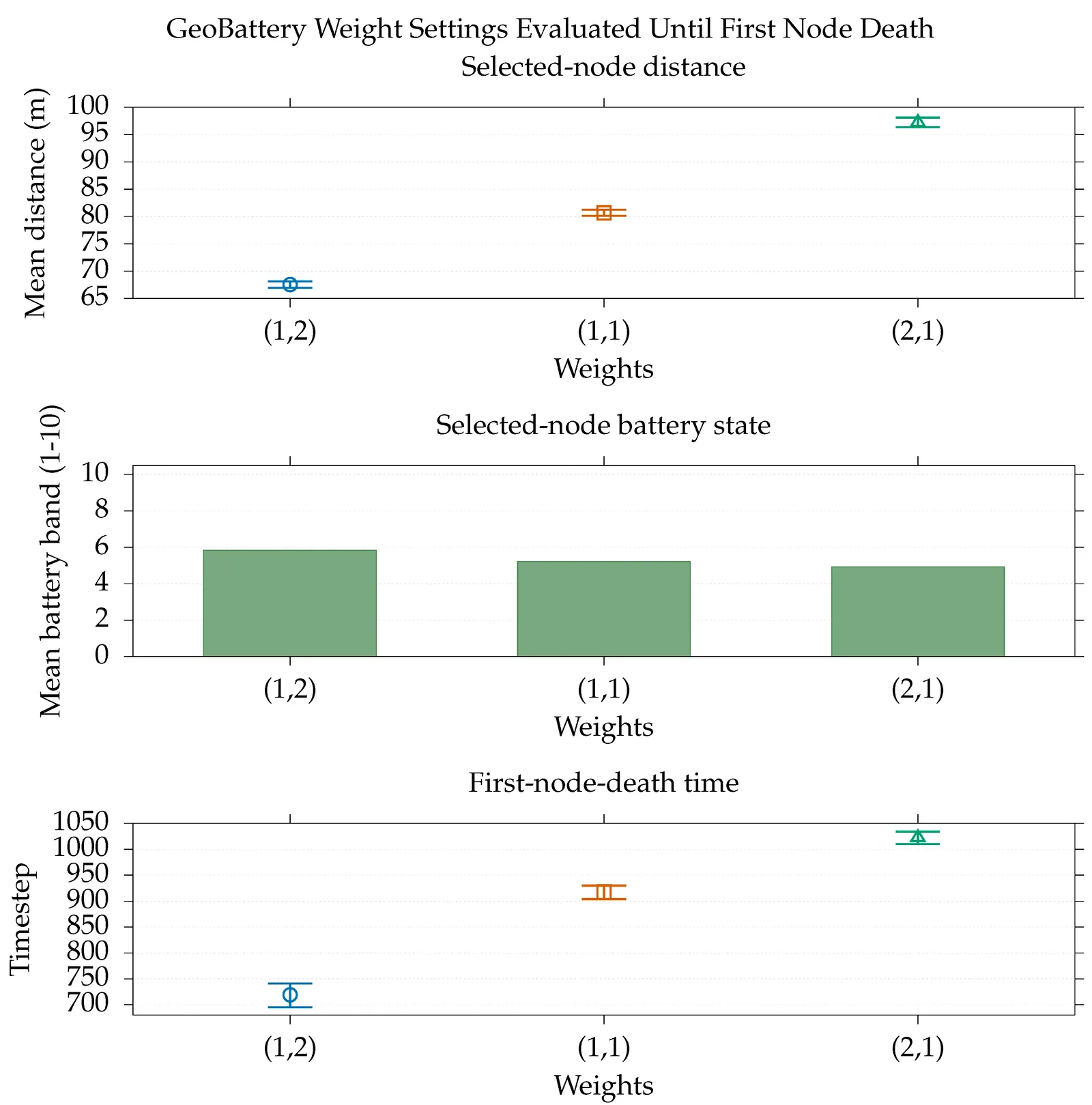
GeoBattery weight settings evaluated until the first node death. Each setting was tested over 30 runs with matched seeds. Symbols distinguish the settings. Each weight pair $\left(w_{b},w_{p}\right)$ gives the battery and proximity weights. Lower distance, a higher battery category, and later first death represent the respective objectives.

Three positive GeoBattery weight settings were evaluated: proximity preference $\left(w_{b},w_{p}\right)=\left(1,2\right)$, equal weights (1,1), and battery preference (2,1). For each setting, 30 independent runs were completed with 1000 generated nodes in a 2000m×2000m area divided into four subareas. Each run was continued until the first node reached zero battery. A synthetic drain of 10 percentage points was applied after each completed measurement, with no idle drain. These rates specify the modeled scenario, while physical energy use requires device measurements. The same node placement, measurement, and initial battery seed triple were used for each replicate across the three settings. Before drain was applied, the selected node’s battery band was recorded for each completed measurement. Ten ordered bands are assigned in the simulator: band 1 covers 0–10%, band 2 covers more than 10% through 20%, and each following band covers the next ten percentage points, up to band 10, which covers more than 90% through 100%. Selected band numbers were averaged over completed measurements within each run, after which the 30 run averages were weighted equally despite their different durations. The resulting average describes an ordinal category, distinct from battery percentage, energy quantity, or depletion rate. Mean target-to-selected-node distance was calculated for each run and then averaged across runs in the same way. A pooled request estimate would instead be weighted by the number of completed requests in each run. The timestep of first node death was also recorded. Results are shown in Figure 5, with 95% percentile bootstrap confidence intervals calculated from 10,000 resamples of the 30 run level results.

The shortest mean placement distance and earliest first depletion were obtained with proximity preference. Under battery preference, farther nodes were selected and first depletion occurred later, with equal weights giving intermediate outcomes (Figure 5). The mean selected battery band decreased as battery weight increased, reflecting the different assignment histories and run durations. All requests were completed up to the terminating timestep, and the maximum feasible GeoBattery score was assigned to every selected node. The same distance ordering was retained in the paired contrasts. Their exploratory intervals were calculated by resampling the 30 shared run IDs 10,000 times. These first death results were obtained with task drain and no idle drain, whereas the fixed-horizon lifetime estimates are reported separately in Section 5.3.

With greater battery weight, more influence was given to the reported state and farther nodes could be selected. When task drain was set to 10 percentage points, first modeled depletion occurred later as assignments were distributed beyond the nearest nodes. The mean selected battery band was affected by repeated task drains, run duration, and the nodes that remained eligible. It summarizes the reports recorded during each run. The results therefore describe the distance accepted in exchange for greater preference for reported reserve, with first death interpreted under the stated depletion assumptions. Energy use and physical battery life require device measurements. Sensing coverage and measurement quality remain to be assessed in the field.

**Figure 6:**
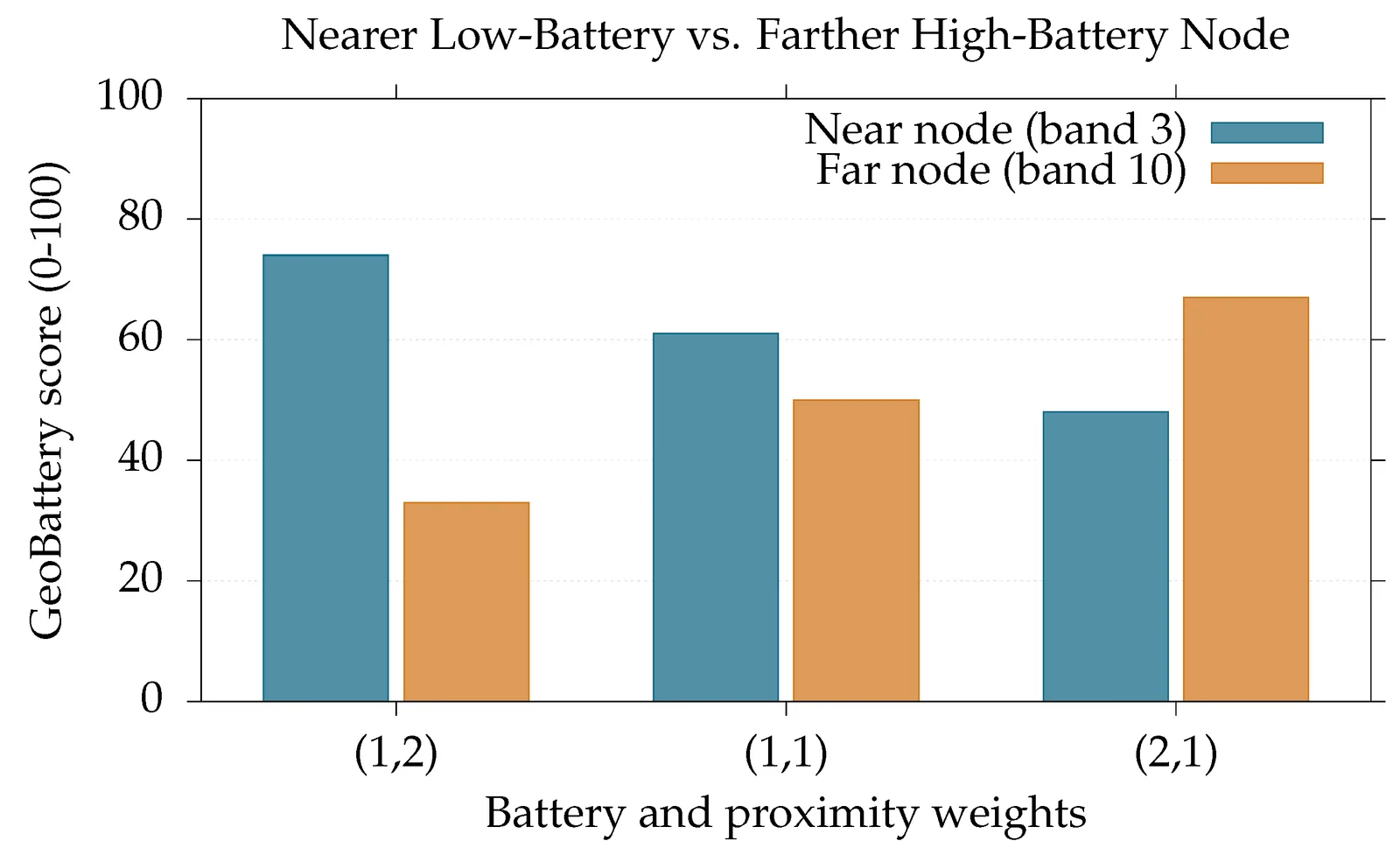
GeoBattery scores in a controlled comparison between a nearer, lower-battery node and a farther, higher-battery node. The scheduler selects the higher-scoring candidate: the near node for (1,2) and (1,1), and the far node for (2,1). Weight pairs are ordered as battery weight followed by proximity weight.

The effect of the configured weights on selection was examined in a controlled framework test where distance and battery state favored different candidates. Two feasible nodes were submitted to the GeoBattery Scheduling Framework plugin: one nearer node reporting battery band 3 and one farther node reporting band 10. A score from 0 to 100 was assigned to each, and the node with the higher score was selected. Proximity preference and equal weights favored the nearer node. Battery preference reversed the ranking and favored the farther node with more reported reserve. This constructed example is separate from the 30-run averages. Scores and selections are shown in Figure 6.

The two-node example shows how the weight ratio affects the distance accepted in exchange for a higher reported battery band. At (2,1), the calculated scores favor the farther node. Different battery bands or locations would change the distance at which this ranking changes. In a cluster, the same calculation is made for every feasible node using its reported state and location.

### 5.2. Measurement Proximity Across Subarea Counts

The default scheduler and GeoBattery were compared with 4, 9, 16, and 25 equal subareas. For each condition, 1000 generated nodes were placed in a 2000m×2000m area, and 30 pairs of node placement and measurement seeds were used. Each run covered ten timesteps, with one measurement per subarea at each timestep. Nodes began at 100% battery. Both measurement drain and idle drain were set to zero so that placement distance could be examined separately. The analyzer confirmed that layouts and target points matched within each scheduler pair. All requested measurements were completed. For each run, the mean Euclidean distance from target to selected node, median request distance, and 95th percentile of request distances were calculated. Scheduler means were obtained by averaging the 30 run means, giving each seed equal weight. The 95% confidence intervals are percentile bootstrap intervals from 10,000 resamples. For each seed, the paired difference was calculated as the default scheduler distance minus the GeoBattery distance.

**Figure 7:**
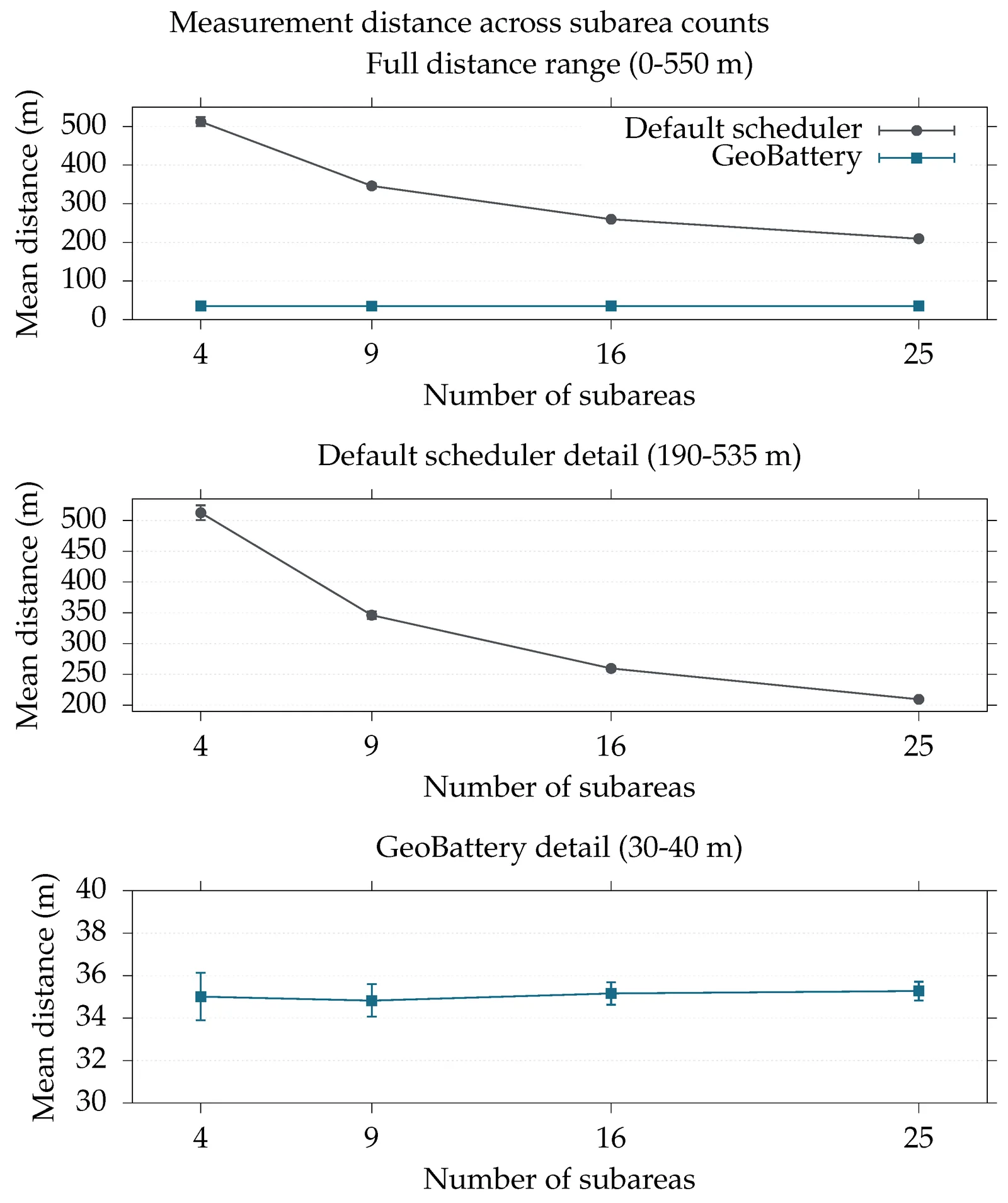
Mean distance from target to selected node across subarea counts. The top panel shows both schedulers on the full 0–550 m scale. The lower panels show the default scheduler (190–535 m) and GeoBattery (30–40 m) separately at a larger scale. Points are means of 30 run means. Error bars show 95% bootstrap confidence intervals. The y axis is labeled in each panel.

As the number of subareas increased, shorter mean placement distances were obtained with the default profile, while GeoBattery’s distances remained similar across the tested range (Table 3, Figure 7). GeoBattery was favored by the paired distance differences at every subarea count, with intervals remaining above zero. A similar pattern was observed in the upper tail of request distances. Longer placements under the default profile were reduced with smaller affinity cells, whereas GeoBattery’s upper-tail distances remained similar. The difference between profiles was thus narrowed by finer grid resolution, although closer nodes continued to be selected by GeoBattery.

Different eligibility rules are applied by the two profiles. Under the default profile, node affinity to the requested subarea is required, while feasible nodes across the cluster are scored by GeoBattery. The distance results are therefore interpreted as a descriptive profile comparison affected by both eligibility and proximity scoring. The same eligible nodes would be needed to isolate the effect of the score. Euclidean distance describes placement relative to the target point, with sensor coverage and physical measurement accuracy treated as separate outcomes.

**Figure 8:**
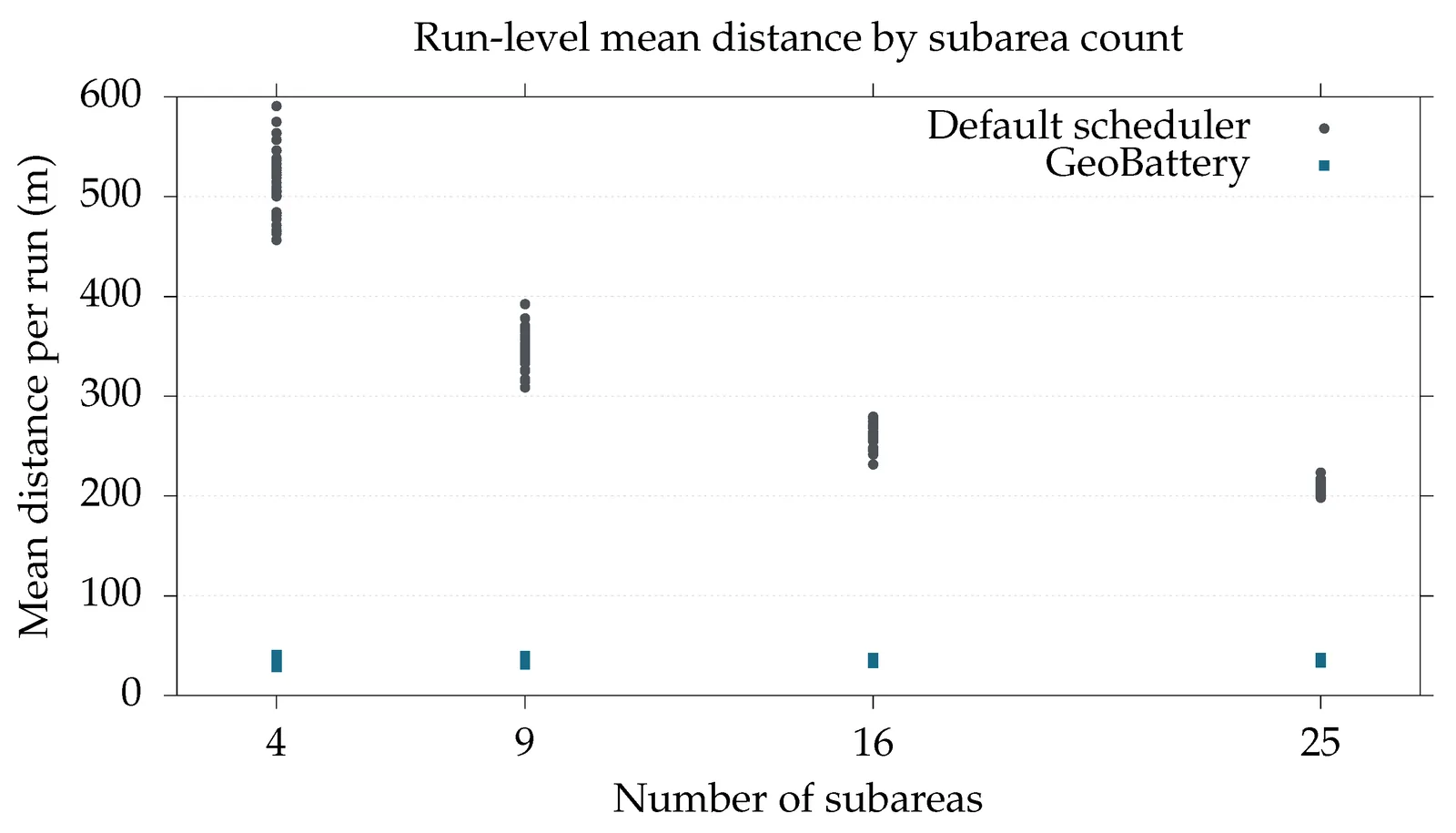
Mean distance from target to selected node in each of the 30 matched runs at every subarea count. Both schedulers share an x position for each count (4, 9, 16, or 25). Color distinguishes the default scheduler from GeoBattery. Each point represents one run.

Figure 8 depicts variation across matched layouts for each subarea count.

### 5.3. Matched-Policy and Sensitivity Analysis over 1000 Timesteps

The evaluation was carried out across 600 simulation runs and 20 settings, with 30 paired replicates per setting. In each run, 1000 nodes were generated in a 2000m×2000m area divided into four equal subareas. Four requests were generated at each timestep over a fixed duration of 1000 timesteps. The same node layout and request locations were supplied to all policies within a matched comparison, so differences could be examined under shared initial and workload conditions.

Five battery and drain scenarios were evaluated:

- *Center condition:* every node was initialized at 100% battery. A task drain of 1 percentage point was applied per completed measurement, and an idle drain of 0.1 percentage points was applied to each node per timestep.
- *Zero idle drain:* the center condition was retained, except that idle drain was set to zero.
- *Lower task drain:* the center condition was retained, except that task drain was set to 0.25 percentage points per completed measurement.
- *Higher task drain:* the center condition was retained, except that task drain was set to 2 percentage points per completed measurement.
- *Unequal initial battery:* the center drain rates were retained, but each node was assigned a starting level by seeded, uniform random selection from the ten ordinal levels 10%, 20%, …, 100%.

Only one battery or drain factor was changed from the center condition in each scenario. Battery percentage was reduced by the specified decimal percentage point drains, with zero as the lower bound. Depletion time was recorded when a node first reached zero. Nodes that remained above zero after timestep 1000 were recorded as surviving to the end of the observation period (right censored).

Three GeoBattery policies were compared: proximity only $\left(w_{b},w_{p}\right)=\left(0,1\right)$, battery only (1,0), and combined (1,1). Kubernetes feasibility and GeoBattery input validation rules were held the same for all policies, although subsequent battery states and candidate sets could diverge as different nodes were selected. Clipping and rounding of the proximity score can also give equal scores to nodes at different distances. A default profile reference was run under the center condition. This result is reported as a descriptive profile comparison because node eligibility is changed by subarea affinity, and matched candidate sets would be required to isolate the scoring effect.

Four additional settings, each changing one factor, were tested for the combined policy under the center condition. Center weights were $\left(w_{b},w_{p}\right)=\left(1,1\right)$, where $w_{b}$ is the battery weight and $w_{p}$ is the proximity weight. The distance scale *D* determines where the proximity contribution reaches zero: it decreases with distance below *D* and is zero at or beyond *D*. The configured default, $D=2000/\sqrt{2}\;m$, is approximately 1414.2 m, half the 2828.4 m diagonal from corner to corner in the 2000m×2000m area. This value was used as the center setting. The alternatives were:

- *Battery-to-proximity weights 3:1: *$\left(w_{b},w_{p}\right)=\left(3,1\right)$, with the default distance scale.
- *Battery-to-proximity weights 1:3: *$\left(w_{b},w_{p}\right)=\left(1,3\right)$, with the default distance scale.
- *Shorter distance scale: *D/2, approximately 707.1 m (one-quarter of the area diagonal), with weights retained at (1,1).
- *Longer distance scale: *2D, approximately 2828.4 m (the full area diagonal), with weights retained at (1,1).

All other parameters of the center condition were held fixed for each setting. Distance scales at half and twice the default were used as bounded multiplicative checks around the configured value. With D/2 (approximately 707.1 m), a zero proximity score is assigned at distances of 707.1 m or more, while with 2D (approximately 2828.4 m), the corresponding boundary is 2828.4 m. Nodes remain eligible beyond these distances. Their battery scores continue to contribute under combined scoring, so sensitivity to both weights and distance scale is examined around the configured defaults. Deployment choices can be informed by workload and device measurements.

Mean placement distance was calculated across completed requests in each run. Completion was defined as the number of completed requests divided by all requested slots, including those left unassigned. Restricted mean lifetime was calculated across the 1000 nodes by averaging each node’s observed time to death or, for survivors, the 1000 timestep cap. Survival at timestep 1000 was defined as the fraction still alive after that timestep. Contrasts were calculated as mean paired differences across the 30 runs, with 95% percentile bootstrap intervals obtained from 10,000 resamples. Both estimates and intervals were based on runs. The protocol, seed construction, event definitions, and raw data are supplied in the Supplementary Material.

Table 4 summarizes the run means for the center condition. Under combined scoring, mean distance fell between the two score ablations, as did request completion. By timestep 1000, every node in every center run had reached zero battery. The default profile reference is shown for context. It was obtained using a different candidate set.

The greater target-to-node distance under the battery policy follows from its zero distance weight. A distant feasible node can be selected whenever its battery band gives it the highest score. Reserve was depleted on every node between requests by idle drain, with task drain also applied to nodes performing measurements. Under the battery policy, task drain was incurred by every node at least once. Later requests were left unassigned when the last available node reached zero before observation ended. Under the proximity policy, nearby nodes were selected repeatedly, concentrating measurement drains on them while some farther nodes received only idle drain. Those nodes remained available later. The final eligible node was depleted later and higher completion was obtained, whereas intermediate distance and completion were obtained under combined scoring. A deployment balance must be chosen according to the relation between geographic placement and reported reserve. Physical energy use and sensing success can be measured on target devices.

Table 5 reports the corresponding lifetime outcomes. Survival after timestep 1000 was zero for all center-policy nodes.

Median node death time was nine timesteps later under proximity-only scoring than under battery-only scoring. This difference reflects how measurement drain was distributed in the simulated workload, rather than a direct measure of network service lifetime. Each node lost 0.1 percentage points per timestep from idle drain, while each completed measurement removed one percentage point from the selected node. Across the 30 runs, every battery-only node was assigned between two and eight measurements. In the 30,000 proximity-only node records, 137 received no measurement, while some received as many as 19. Thus, measurement drain was spread across more nodes under battery-only scoring, while a portion of the proximity-only nodes incurred idle drain alone. Since the battery-only policy ranks nodes by reported battery band and does not optimize fleet lifetime, this broader exposure to measurement drain is consistent with its earlier median node death time. The mean restricted lifetimes remained close, and all nodes in the center condition reached zero by timestep 1000. Median node death time therefore describes a typical node in this workload and does not identify when the network can no longer serve requests.

Paired differences in distance and completion are given in Table 6. The intervals for these contrasts remained on one side of zero, supporting the direction of the observed trade-offs across matched runs.

The contrasts in Table 6 show the trade-off between distance and completion. Relative to the proximity policy, combined scoring increased distance slightly and lowered completion in exchange for a battery preference. Relative to the battery policy, it reduced distance substantially and raised completion modestly. Restricted mean lifetimes were similar, and survival at the end of observation was the same. Placement and completion were affected mainly by which nodes remained available near request points, while changes in mean modeled lifetime were small. Under these conditions, adding the battery preference to proximity scoring incurred a distance and completion cost with a small increase in mean modeled lifetime. This cost can be assessed against the deployment’s acceptable placement distance and required request completion.

**Figure 9:**
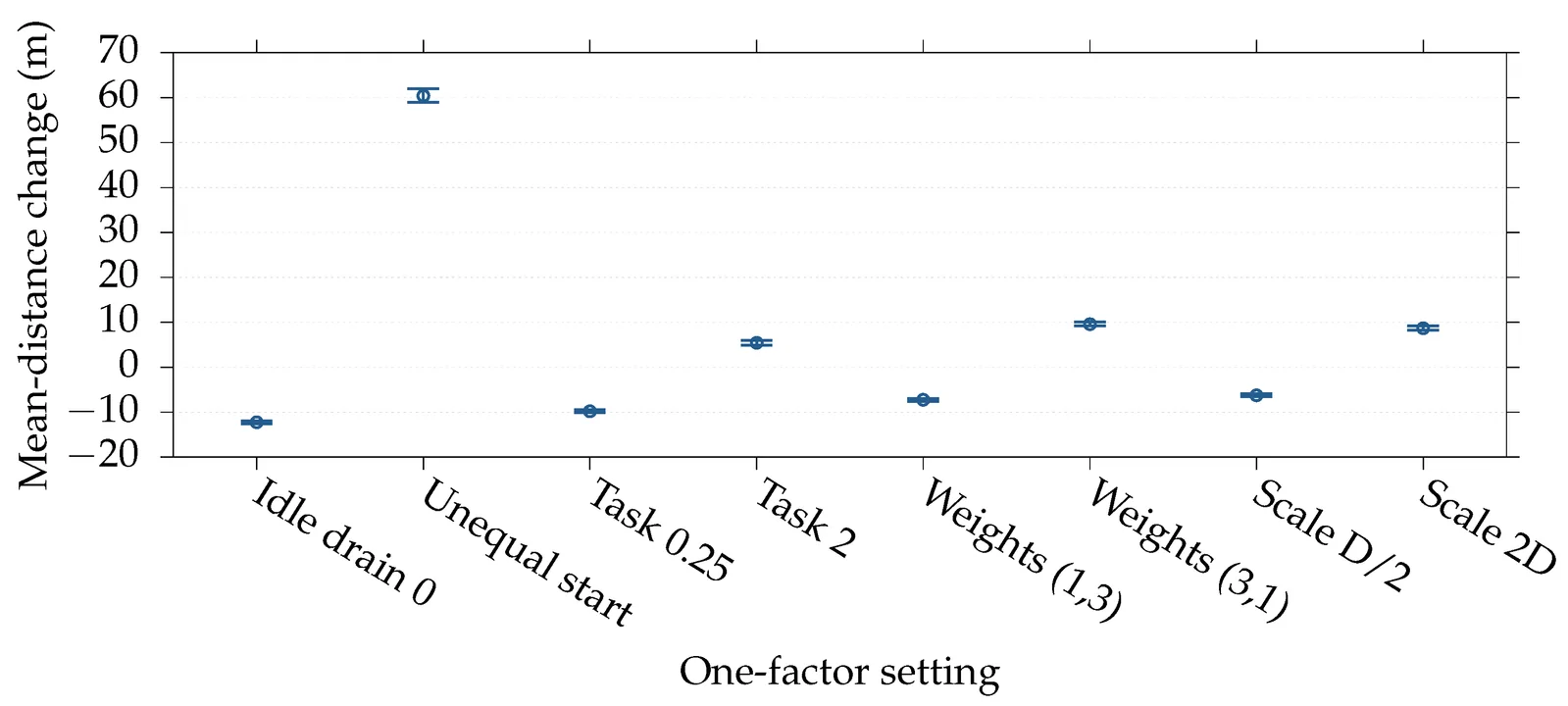
Paired change in mean distance from target to selected node for each single factor setting relative to the center combined policy. Points are mean differences across runs. Capped error bars are 95% paired percentile bootstrap intervals from 30 replicate IDs. Negative values denote shorter mean distance than the center setting.

**Figure 10:**
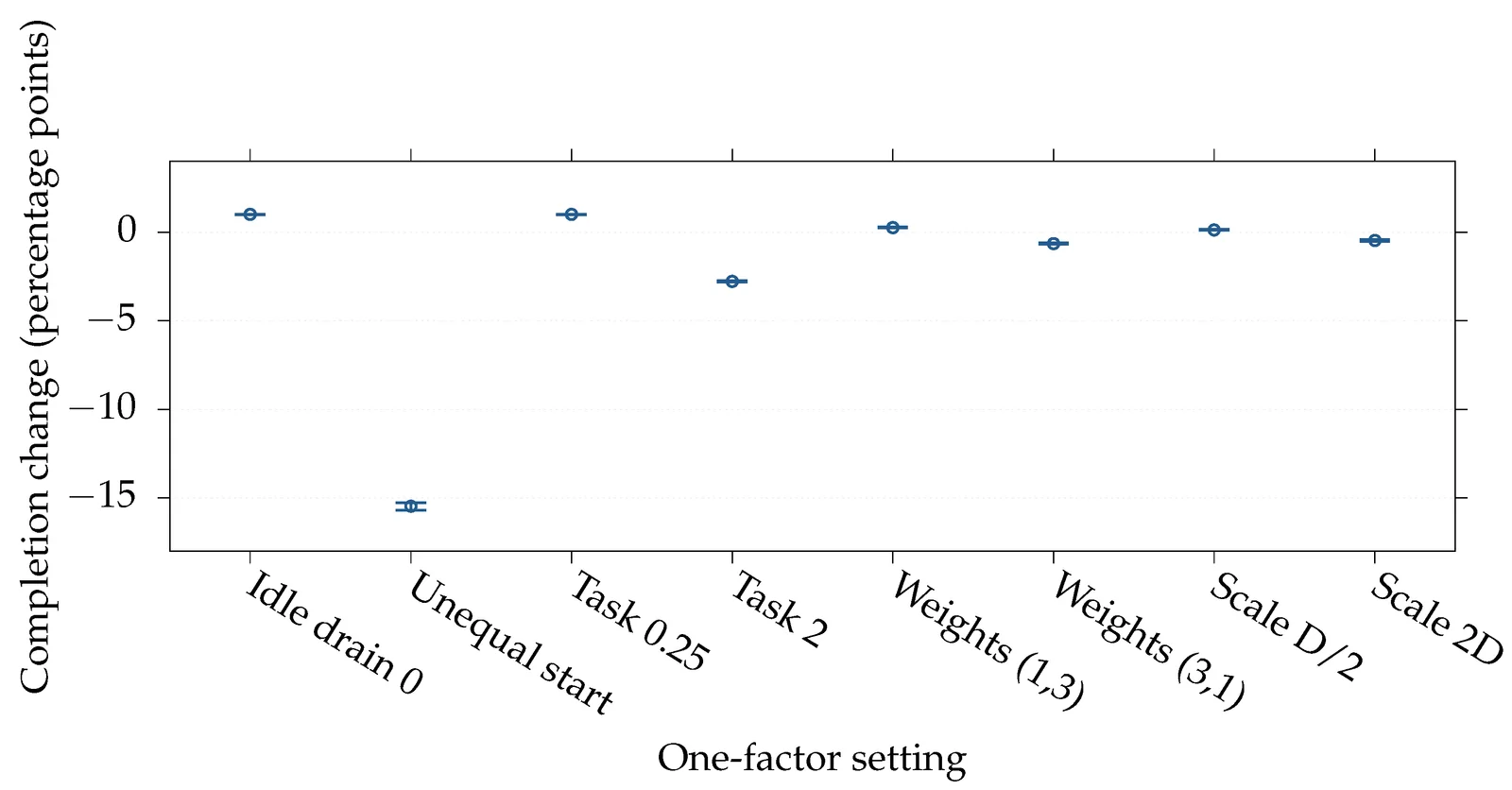
Paired change in request completion, in percentage points, for each single factor setting relative to the center combined policy. Points are mean differences across runs. Capped error bars are 95% paired percentile bootstrap intervals from 30 replicate IDs. Negative values denote lower completion than the center setting.

Figure 9 and Figure 10 show paired changes in distance and completion from the center setting with combined scoring.

The drain and initial state settings also have a direct resource interpretation. When idle drain was removed or task drain was reduced, more modeled reserve remained between and during assignments. More nodes were then eligible for later requests. Both settings increased completion and restricted mean lifetime relative to the center condition. Higher task drain consumed more reserve for each completed measurement, shortened the time during which nodes could serve requests, and reduced completion. With unequal initial levels, some nodes began with less reserve and fewer could sustain repeated service. Both completion and restricted mean lifetime decreased. The percentage point drains are synthetic inputs. Energy costs must be measured on calibrated devices. With no idle drain, all nodes remained above zero through timestep 1000, so their recorded lifetimes represent survival through the observation period. These sensitivity findings apply to the battery bands and workloads tested. Alternative mappings, device calibration, and settings for specific workloads remain to be examined.

With weights (1,3) or a halved distance scale, mean distance decreased and completion improved slightly. With weights (3,1) or a doubled distance scale, farther nodes could compete, mean distance increased, and completion decreased. Drain scenarios produced larger changes than the score settings, particularly when initial reserve was unequal or task drain was higher. The modeled operating conditions therefore affected outcomes more than the tested weight changes. These findings apply to the specified node distribution, request pattern, score quantization, and drain assumptions. Other workloads may produce different outcomes. Workload and battery measurements can guide the choice of deployment settings.

Fleet-level changes are listed in Table 7. With no idle drain, every node remained available throughout observation. Lower task drain increased mean modeled lifetime, although all nodes still reached zero by timestep 1000. Higher task drain and unequal initial reserve shortened lifetime. Changes to score weights and distance scale produced small lifetime differences, with all nodes depleted by the end of observation. Under this workload with positive idle drain, assignment patterns affected outcomes more than the overall depletion endpoint.

Most first zero events across the campaign were attributed to idle drain, with the remainder attributed to measurement drain. Background consumption can deplete nodes even when few measurement tasks are assigned in a battery-powered deployment, while a workload-dependent cost is added by tasks on the selected nodes. Surviving nodes all came from settings with no idle drain and were right-censored at the end of observation. Every other run had exhausted its modeled batteries by that time, and requests were left unassigned when a feasible node was unavailable. These results describe depletion under the stated assumptions. Device-level instrumentation is needed to measure energy in joules, battery capacity, and physical service life. The center comparison concerns placement and request outcomes under the same model.

### 5.4. Simulator Cost Across Node Counts

The size sweep comprised 240 simulator processes. The cost measurements apply to the simulator configuration used in this experiment. Computational cost for the lifetime evaluation in Section 5.3 remains to be measured with its own execution configuration. Source code and execution configurations are supplied in the Supplementary Material.

Selection latency, total simulator CPU time, and process peak resident set size (RSS) were measured at 200, 500, 1000, and 2000 generated nodes for the default and GeoBattery profiles. For each profile and size, 30 runs with matched seeds were completed, with 400 measured requests per run. Four subareas, equal GeoBattery weights, and zero battery drain were held fixed. Selection latency covers the selection operation, including the node snapshot, feasibility checks, scoring, and tie resolution. Total simulator CPU time covers startup, initialization, all requests, and output. RSS is the process high water mark sampled when simulation ended. Each plotted value is the equally weighted mean of 30 run summaries. Error bars show 95% percentile bootstrap confidence intervals from 10,000 resamples of the run-level data. Execution settings, timing checks, and reproduction steps are provided in the Supplementary Material.

**Figure 11:**
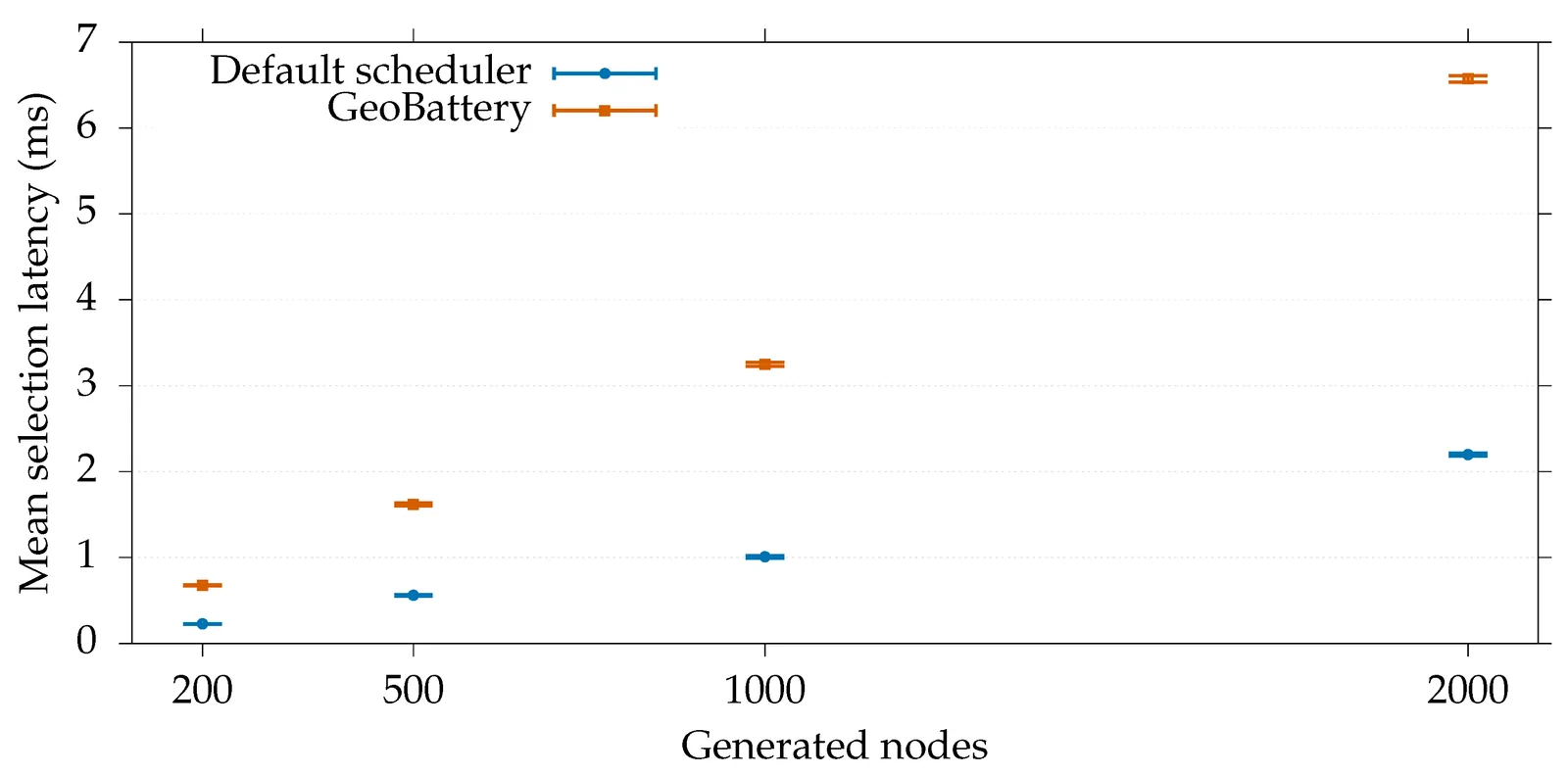
Mean selection latency across generated node counts. Points are means of 30 request means, one per run. Bars show 95% bootstrap confidence intervals across runs. The profiles use different candidate eligibility rules.

Mean selection latency rose with node count for both profiles, with GeoBattery taking longer throughout the tested range (Figure 11). At each selection, more nodes had to be considered in the larger generated cluster. GeoBattery also checks battery conditions and calculates a score based on battery state and proximity. In this workload, subarea affinity restricted the default profile to nodes in the requested subarea, while GeoBattery considered nodes across the cluster. The latency difference therefore reflects both the number of candidates and policy work, and cannot be attributed exclusively to GeoBattery’s scoring mechanism. The added cost of GeoBattery could be isolated by using the same candidates for both profiles.

**Figure 12:**
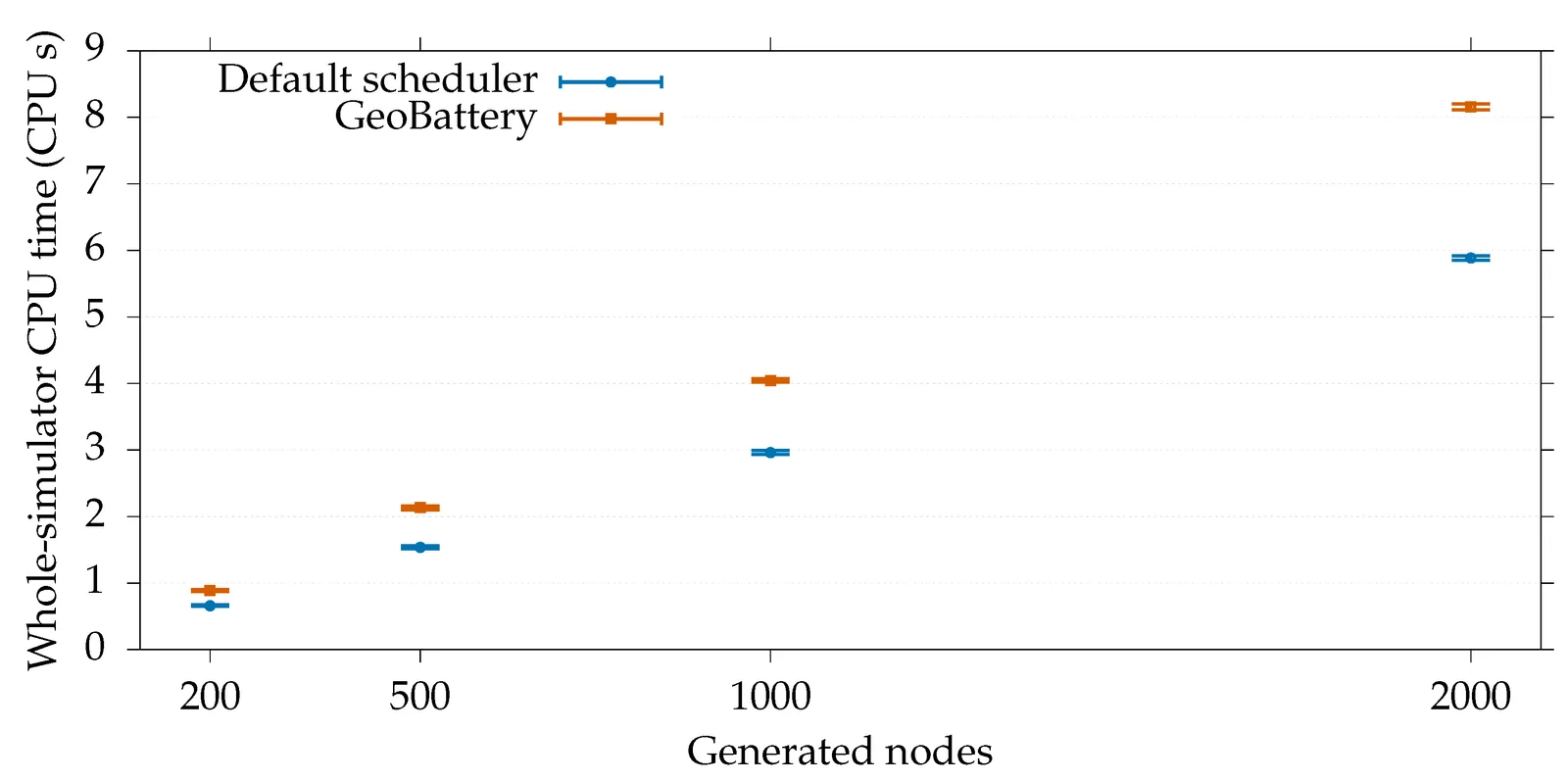
Whole-simulator CPU time per process, including initialization, requests, and output. User and system CPU time are summed over all threads. Points are 30-run means with 95% run-bootstrap confidence intervals.

Total simulator CPU time also rose with cluster size and was higher for GeoBattery at each tested size (Figure 12). These totals include user and system CPU time across all simulator threads. Larger node sets required more initialization and node evaluation work, and a larger feasible set was validated and scored by GeoBattery. As with selection latency, the CPU difference cannot be attributed exclusively to GeoBattery’s scoring mechanism because the profiles use different candidate sets. The CPU results describe the cost of the complete simulator process, while selection latency describes the individual placement operation.

**Figure 13:**
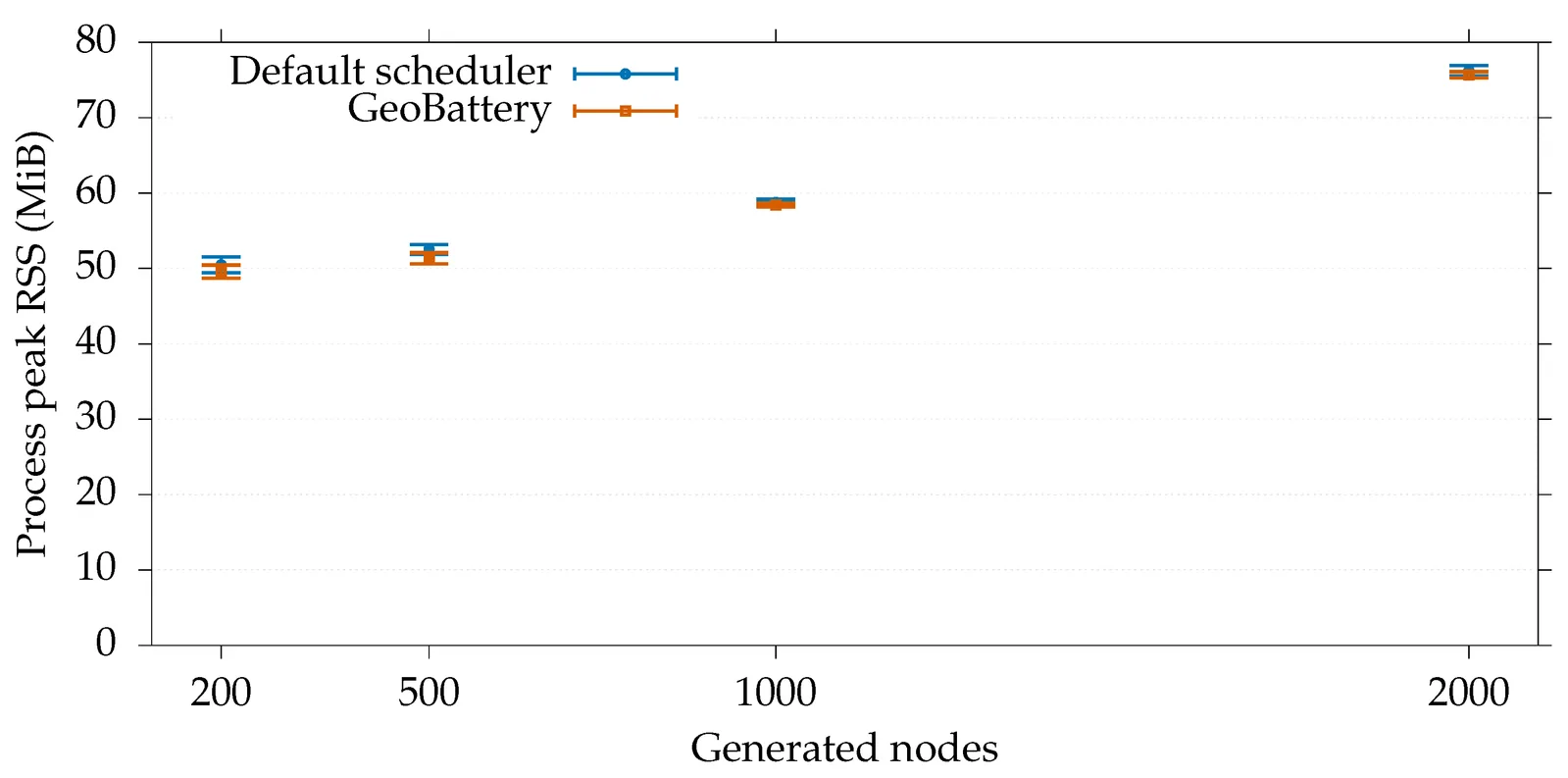
Process peak resident set size sampled at simulation completion, in MiB. Points are 30-run means with 95% run-bootstrap confidence intervals.

Peak process RSS rose with node count and remained similar between profiles, with overlapping confidence intervals at every tested size (Figure 13). Both profiles use the same generated graph, framework, and Go runtime structures, which all contribute to process memory. The observed memory results were therefore similar despite the differences in selection latency and total CPU time. These process-level measurements describe the combined memory footprint of the simulator and its scheduling components.

## 6. Discussion

Placement and completion were affected by the relative weights assigned to the two inputs. Under the proximity policy, task drains were concentrated near frequently requested points because nearby nodes were selected repeatedly. Some more distant nodes received only idle drain and remained available for later requests. Under the battery policy, scores were determined entirely by the current battery band, with zero weight assigned to target proximity. Distant workers could therefore be selected. Task drain was incurred by every node at least once, in addition to the background loss imposed by idle drain, and the last eligible node was depleted earlier. With both inputs, greater distance was accepted than under proximity scoring, but substantially shorter placements were obtained than under battery scoring. Completion was also intermediate. A suitable balance for a physical deployment can be determined through device and workload measurements.

With positive idle drain in the center condition, all nodes under the three matched GeoBattery policies had reached zero by the end of observation. The same was true of the separate default profile reference. With no idle drain, every node remained above zero and was right censored at the observation endpoint. Center restricted mean lifetimes were similar across policies within the same observation window. Idle drain represents background consumption, which can limit later service even when few tasks are assigned. Physical battery endurance can be measured using calibrated percentage point rates under a defined workload. Since every node in the center condition reached the modeled battery floor, the comparison describes placement and request outcomes under the synthetic depletion model.

Workload effects can be distinguished from score changes through the sensitivity results. More reserve was retained for later requests when idle or task drain was lower. With higher task drain or unequal starting levels, fewer nodes remained available to serve the workload. The balance between spatial preference and battery state was set by proximity weight and distance scale: closer workers were favored by greater proximity weight, while farther nodes could compete under greater battery weight or a longer distance scale. Completion was also affected by request order, changes in battery reports, and score ties. Other layouts or task patterns may give different results. Since one factor was changed at a time, these tests describe responses around the configured settings, with bootstrap intervals representing variation across the 30 paired runs under the synthetic model. They are bounded one-factor checks, not an exhaustive parameter optimization, and they do not identify an optimal setting. Device measurements are needed to calibrate drain rates, ordinal bands, and physical battery variation. Alternative band mappings and calibration for particular devices and workloads remain to be tested. Band order is preserved by the linear mapping, but empirical calibration is required before equal increments can be related to energy.

The placement study across 4, 9, 16, and 25 subareas illustrates a practical choice between geographic affinity and ranking by target point. With affinity, placement distance is affected by the chosen cell size. When the target point is used in scoring, a similar mean distance was obtained across the grid sizes tested. Sensor reach, coverage, and measurement accuracy require field measurements.

In the node count sweep, decision time and total computation increased with the number of nodes examined. Process memory remained similar between profiles at the tested sizes. Longer selection cycles may delay pending work, and CPU and memory are shared with other scheduler processes. Selection time, simulator computation, and process memory were measured within the simulator process. An evaluation in a live cluster can include API server interactions, queueing, network communication, binding, and cluster contention. Since the profiles apply different eligibility rules, the measured overhead reflects both candidate count and policy work and cannot be attributed exclusively to GeoBattery’s scoring mechanism. The sweep included up to 2000 nodes. Larger clusters remain to be examined.

Modeled energy use and application interference are addressed in KEIDS, while placement is informed by energy and greenness indicators in CODECO [8]. Runtime telemetry and workload performance are used in Chronos for fog and IoT scheduling [9]. Processing and network delay are addressed in DACS. Network infrastructure is considered by Santos et al. for fog resource provisioning [4], and topology and service level objectives are addressed in Polaris across cloud, edge, and IoT clusters [7]. In CosmoSpan, workload descriptions are used to schedule and remove workloads across federated clusters [10]. Placement in GeoBattery is based on a fresh ordinal battery report and distance from a target point. Internal policy comparisons are reported here alongside source-verified context, while practical performance across these systems would require assessment with a shared implementation and workload.

GeoBattery is intended for sensing pods with finite target points in a shared local Cartesian frame and nodes reporting a unique, fresh ordinal battery level. Feasibility is established by the standard Kubernetes resource, affinity, taint, and toleration checks, after which retained nodes are ranked according to the configured proximity and battery preferences. Both placement preferences can be set by the operator. Point distance represents spatial separation, while sensor reachability and task success depend on the application and device. Sensor acquisition, battery reporting, task energy reservation, and network aware placement are handled by separate functions. The scheduler was evaluated in process using homogeneous generated nodes and synthetic percentage point drains. Findings are limited to the workloads, initial states, drain rates, score settings, eligibility rules, and 1000 timestep observation window tested. In the lifetime summaries, nodes above zero at timestep 1000 were right-censored and counted as surviving through the window, with restricted mean lifetime referring to the same observation period. Device-calibrated discharge and sensing outcomes can be evaluated alongside queueing, API, network, binding, and contention costs in a live cluster.

## 7. Conclusions and Future Work

GeoBattery was implemented as an optional Kubernetes Scheduling Framework policy for ranking feasible nodes by distance from a target point and a fresh ordinal battery report. The effects of both inputs were examined in the evaluation. Nearby nodes were favored when greater weight was assigned to proximity, whereas a farther node with more reported reserve could be selected under greater battery weight. When task consumption was added to background depletion, the time for which nodes remained available was also affected by assignment patterns. Intermediate distance and completion were obtained with both inputs. In the separate subarea experiment, shorter target-to-node distances were obtained with GeoBattery than with the default profile. Since different candidate sets were used, the comparison reflects both eligibility and scoring.

In the 1000-timestep campaign, the distance and completion trade-off was observed under matched conditions. With combined scoring, workers were placed farther from the target and fewer requests were completed than under proximity scoring. Shorter placements and higher completion were obtained relative to battery scoring. Restricted mean lifetimes in the center condition differed little relative to the 1000 timestep observation period, although the measured outcomes were affected by the sensitivity settings. Deployment parameters can be informed by workload and battery measurements. These findings concern simulated scheduler behavior, with physical energy use, battery endurance, and sensor quality remaining to be assessed through calibrated devices and field measurements.

Mean selection latency, total simulator CPU time, and peak process RSS were measured for 200–2000 generated nodes. The profiles applied different eligibility rules, so the comparison reflects both candidate count and policy work. API server, queueing, network, and binding costs, as well as a wider range of cluster sizes, can be examined in a live cluster. Profiling and optimization may reduce selection latency and CPU time while preserving placement behavior. A source verified comparison with energy-oriented and other edge schedulers is provided in the related work section. Further evaluation can use common workloads, alternative battery band mappings, calibration for devices and workloads, and physical device or live cluster settings.

## Figures and Tables

**Table 1:** GeoBattery’s runtime interface and validation roles.

Data	Kubernetes Field	Validation or Use
Target x,y	Pod annotations iot.geobattery/x-m, iot.geobattery/y-m	Finite values in meters
Node x,y	Node labels iot.example.org/x-m, iot.example.org/y-m	Finite values in the shared frame
Battery level	Node conditions BATTERYLEVEL1–BATTERYLEVEL10	Unique True level, remaining present levels False
Heartbeat	Active battery condition	Age in [0, τ] at cycle time
Weights and scale	Plugin configuration	Finite weights, positive sum, positive scale

**Table 2:** GeoBattery request, node, and policy inputs.

Input	Location	Role in the Model
Target coordinates $\left(x_{p},y_{p}\right)$	iot.geobattery/x-m, iot.geobattery/y-m	Define the requested point in local meters
Node coordinates $\left(x_{i},y_{i}\right)$	iot.example.org/x-m, iot.example.org/y-m	Define the worker location in the same local frame
Battery level and heartbeat	Node conditions	Provide a fresh ordinal state used for validation and scoring
Battery/proximity weights	Scheduler configuration	Set the relative contribution of each preference
Proximity scale *D*	Scheduler configuration	Normalize distance into a score contribution
Maximum battery age τ	Scheduler configuration	Bound the acceptable age of the active heartbeat

**Table 3:** Mean distance from target to node and paired differences between the default scheduler and GeoBattery across subarea counts. Values are in meters. Intervals are 95% bootstrap confidence intervals across 30 matched seeds.

Subareas	Default Scheduler Mean (95% CI)	GeoBattery Mean (95% CI)	Paired Distance Difference (95% CI)
4	512.46 (500.66–524.68)	35.01 (33.90–36.13)	477.44 (465.44–489.38)
9	346.08 (339.69–352.68)	34.83 (34.07–35.60)	311.26 (304.91–317.80)
16	259.79 (255.54–263.81)	35.16 (34.63–35.69)	224.63 (220.41–228.64)
25	209.44 (207.48–211.44)	35.28 (34.83–35.72)	174.16 (172.26–176.06)

**Table 4:** Mean distance and request completion in the center condition across 30 runs. Each entry gives the mean and 95% percentile bootstrap interval across runs.

Policy	Mean Distance (m) [95% CI]	Completion (%) [95% CI]
Proximity-only (0,1)	37.437 [37.177, 37.690]	99.9675 [99.9358, 99.9917]
Battery-only (1,0)	1042.850 [1039.073, 1046.716]	98.0000 [98.0000, 98.0000]
Combined (1,1)	47.868 [47.523, 48.227]	98.9958 [98.9767, 99.0117]
Default-profile reference	521.509 [520.047, 522.909]	99.1867 [99.1592, 99.2158]

**Table 5:** Restricted mean modeled lifetime, median node death time, and survival after timestep 1000 in the center condition. Restricted mean lifetime values are means across 30 runs with 95% percentile bootstrap intervals across runs. Median node death times are descriptive medians across the 30,000 observed node death times for each policy.

Policy	Restricted Mean Lifetime (Steps) [95% CI]	Median Node Death Time (Steps)	Survival After Timestep 1000 (%)
Proximity-only (0,1)	960.757 [960.740, 960.773]	969	0
Battery-only (1,0)	961.296 [961.296, 961.296]	960	0
Combined (1,1)	961.135 [961.118, 961.152]	960	0
Default-profile reference	960.890 [960.874, 960.905]	960	0

**Table 6:** Distance and completion effects for combined scoring relative to each matched score ablation in the center condition. Values are mean paired differences with 95% paired percentile bootstrap intervals. Neither contrast changed the proportion surviving after timestep 1000.

Paired Contrast	Distance Difference (m) [95% CI]	Completion Difference (Percentage Points) [95% CI]
Combined − proximity-only	+10.430 [+10.065, +10.804]	−0.972 [−1.000, −0.939]
Combined − battery-only	−994.983 [−998.907, −991.238]	+0.996 [+0.977, +1.012]

**Table 7:** One-factor changes in restricted mean modeled lifetime and the proportion of nodes surviving through timestep 1000 relative to the center combined-policy condition. Values are mean paired differences with 95% paired percentile-bootstrap intervals across 30 run IDs.

One-Factor Setting	Restricted Mean Lifetime Difference (Steps)	Surviving Nodes After Timestep 1000 Difference (Percentage Points)
Zero idle drain	+38.865 [+38.848, +38.881]	+100 [+100, +100]
Unequal initial battery	−444.113 [−447.060, −441.117]	0 [0, 0]
Task drain 0.25	+29.179 [+29.161, +29.196]	0 [0, 0]
Task drain 2	−35.991 [−36.025, −35.956]	0 [0, 0]
Weights (1,3)	−0.149 [−0.170, −0.128]	0 [0, 0]
Weights (3,1)	+0.099 [+0.077, +0.119]	0 [0, 0]
Distance scale D/2	−0.079 [−0.097, −0.061]	0 [0, 0]
Distance scale 2D	+0.073 [+0.051, +0.095]	0 [0, 0]

## Data Availability

The simulator and scheduler source code, dependency locks, experiment configurations and seeds, raw outputs and metadata, aggregate data, analysis scripts, source/data manifest, and reproduction instructions are supplied in the Supplementary Material. Public release can be considered separately. Environment provenance and the scope of the reproduction checks are documented in the supplementary experiment record.

## Supplementary Materials

The following supporting information can be downloaded at https://www.mdpi.com/article/10.3390/s26217003/s1, Figure S1: Local Cartesian representation of the target point and two worker nodes; Figure S2: GeoBattery scheduling model from pod request to node selection; Figure S3: GeoBattery’s Scheduling Framework path; Figure S4: Illustrative score trade-off under equal battery and proximity weights; Figure S5: GeoBattery weight settings evaluated until the first node death; Figure S6: GeoBattery scores in a controlled comparison between a nearer, lower-battery node and a farther, higher-battery node; Figure S7: Mean distance from target to selected node across subarea counts; Figure S8: Mean distance from target to selected node in each of the 30 matched runs at every subarea count; Figure S9: Paired change in mean distance from target to selected node for each single factor setting relative to the center combined policy; Figure S10: Paired change in request completion, in percentage points, for each single factor setting relative to the center combined policy; Figure S11: Mean selection latency across generated node counts; Figure S12: Whole-simulator CPU time per process, including initialization, requests, and output; Figure S13: Process peak resident set size sampled at simulation completion, in MiB; Table S1: GeoBattery’s runtime interface and validation roles; Table S2: GeoBattery request, node, and policy inputs; Table S3: Mean distance from target to node and paired differences between the default scheduler and GeoBattery across subarea counts; Table S4: Mean distance and request completion in the center condition across 30 runs; Table S5: Restricted mean modeled lifetime, median node death time, and survival after timestep 1000 in the center condition; Table S6: Distance and completion effects for combined scoring relative to each matched score ablation in the center condition; Table S7: One-factor changes in restricted mean modeled lifetime and the proportion of nodes surviving through timestep 1000 relative to the center combined-policy condition.
