# Supplementary material for: Geographic and Battery-Aware Kubernetes Scheduling for IoT Sensing
Source: Sensors (Basel). 2026 Nov 5;26(21):7003. doi: 10.3390/s26217003 (PMC13766508; doi:10.3390/s26217003)
Supplement: Supplementary file 1 [file sensors-26-07003-s001.zip › figure_S1.pdf]

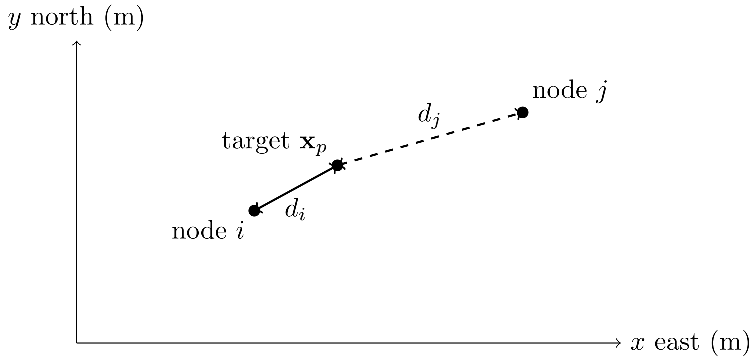


Figure S1: Local Cartesian representation of the target point and two worker nodes. The solid line shows the distance from the target to node $i$, and the dashed line shows the distance from the target to node $j$. Sensing-coverage regions and maximum-range boundaries are separate application constraints.
