# Supplementary material for: Geographic and Battery-Aware Kubernetes Scheduling for IoT Sensing
Source: Sensors (Basel). 2026 Nov 5;26(21):7003. doi: 10.3390/s26217003 (PMC13766508; doi:10.3390/s26217003)
Supplement: Supplementary file 1 [file sensors-26-07003-s001.zip › figure_S2.pdf]

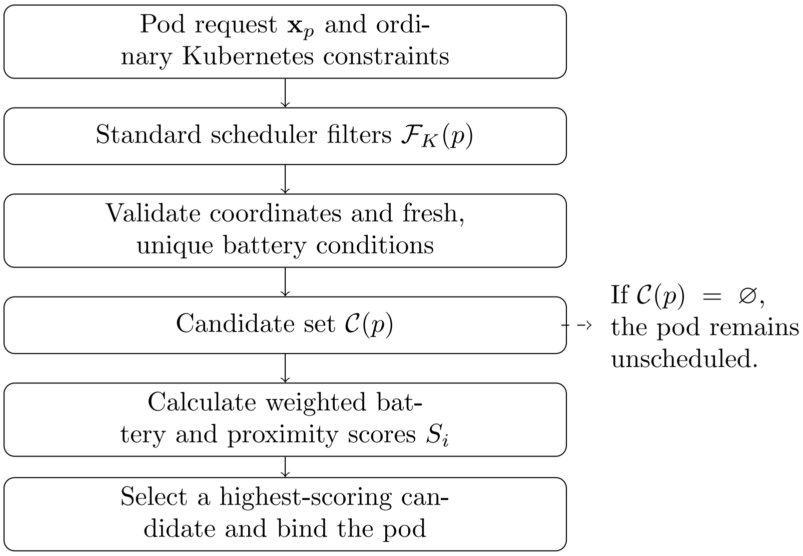

Figure S2: GeoBattery scheduling model from pod request to node selection. Solid arrows show the scheduling flow; the dashed arrow points to the note that the pod remains unscheduled if there are no candidates. The custom policy validates and scores candidates within the standard Kubernetes scheduling cycle.
