# Supplementary material for: Geographic and Battery-Aware Kubernetes Scheduling for IoT Sensing
Source: Sensors (Basel). 2026 Nov 5;26(21):7003. doi: 10.3390/s26217003 (PMC13766508; doi:10.3390/s26217003)
Supplement: Supplementary file 1 [file sensors-26-07003-s001.zip › figure_S3.pdf]

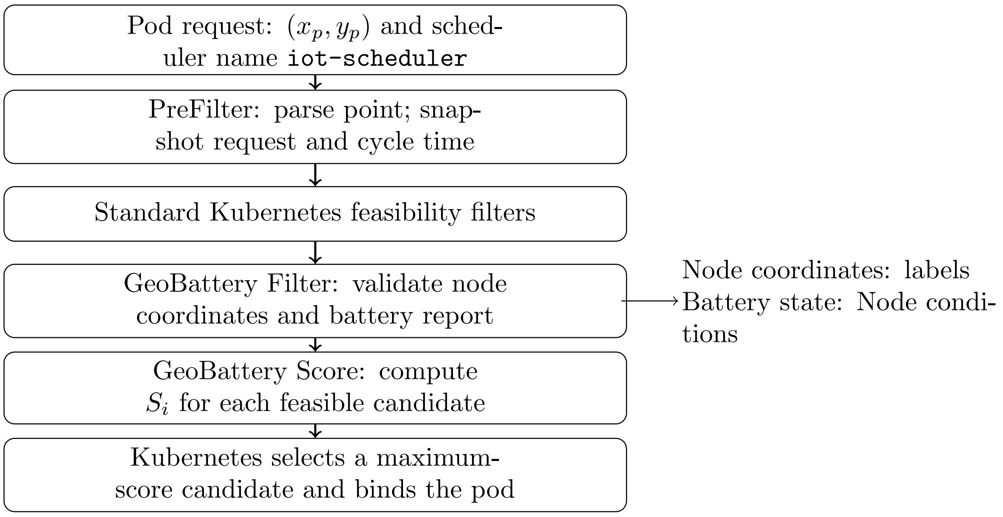

Figure S3: GeoBattery's Scheduling Framework path. Standard filters and plugin validation establish the feasible set before score-based node selection.
