# Supplementary material for: Geographic and Battery-Aware Kubernetes Scheduling for IoT Sensing
Source: Sensors (Basel). 2026 Nov 5;26(21):7003. doi: 10.3390/s26217003 (PMC13766508; doi:10.3390/s26217003)
Supplement: Supplementary file 1 [file sensors-26-07003-s001.zip › figure_S4.pdf]

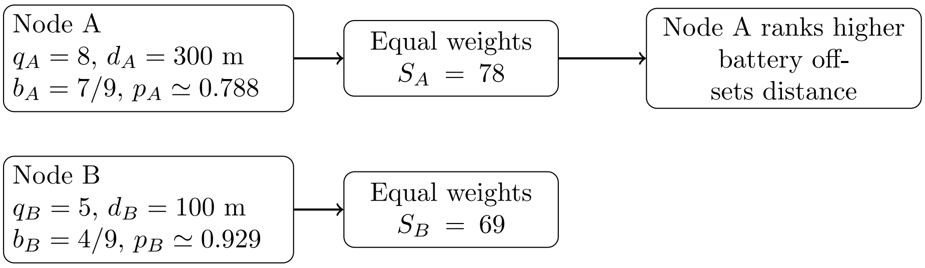

Figure S4: Illustrative score trade-off under equal battery and proximity weights. Node B is closer, while Node A's higher battery band produces the larger rounded score. Values are calculated from the stated score model.
