# Supplementary material for: Geographic and Battery-Aware Kubernetes Scheduling for IoT Sensing
Source: Sensors (Basel). 2026 Nov 5;26(21):7003. doi: 10.3390/s26217003 (PMC13766508; doi:10.3390/s26217003)
Supplement: Supplementary file 1 [file sensors-26-07003-s001.zip › figure_S5.pdf]

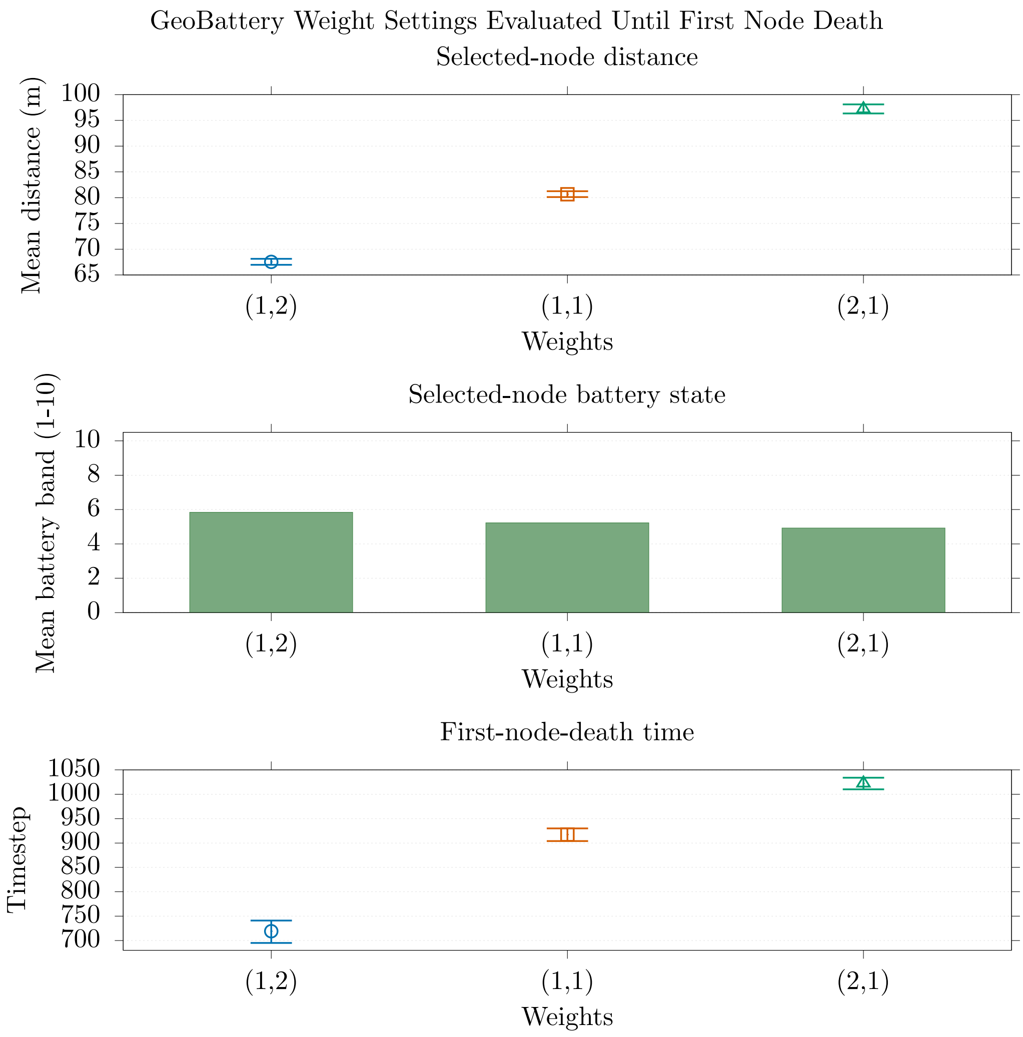

Figure S5: GeoBattery weight settings evaluated until the first node death. Each setting was tested over 30 runs with matched seeds. Symbols distinguish the settings. Each weight pair (w_b, w_p) gives the battery and proximity weights. Lower distance, a higher battery category, and later first death represent the respective objectives.
