# Supplementary material for: Geographic and Battery-Aware Kubernetes Scheduling for IoT Sensing
Source: Sensors (Basel). 2026 Nov 5;26(21):7003. doi: 10.3390/s26217003 (PMC13766508; doi:10.3390/s26217003)
Supplement: Supplementary file 1 [file sensors-26-07003-s001.zip › figure_S6.pdf]

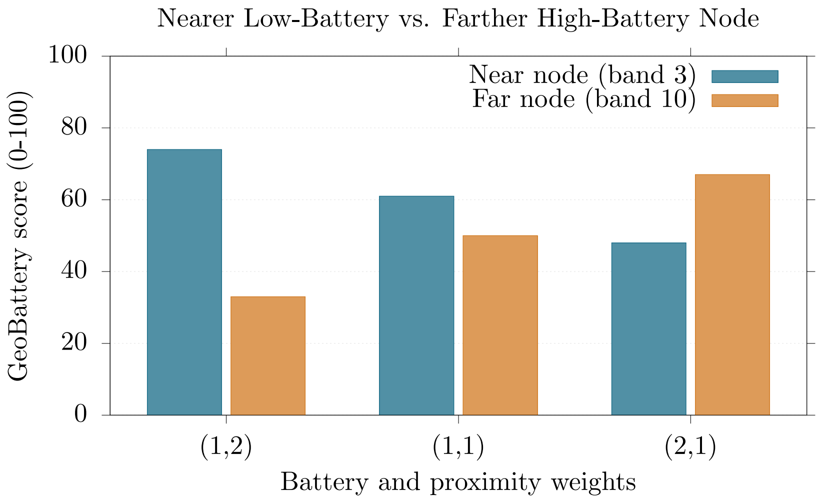

Figure S6: GeoBattery scores in a controlled comparison between a nearer, lower-battery node and a farther, higher-battery node. The scheduler selects the higher-scoring candidate: the near node for $(1, 2)$ and $(1, 1)$, and the far node for $(2, 1)$. Weight pairs are ordered as battery weight followed by proximity weight.
