# Supplementary material for: Geographic and Battery-Aware Kubernetes Scheduling for IoT Sensing
Source: Sensors (Basel). 2026 Nov 5;26(21):7003. doi: 10.3390/s26217003 (PMC13766508; doi:10.3390/s26217003)
Supplement: Supplementary file 1 [file sensors-26-07003-s001.zip › figure_S7.pdf]

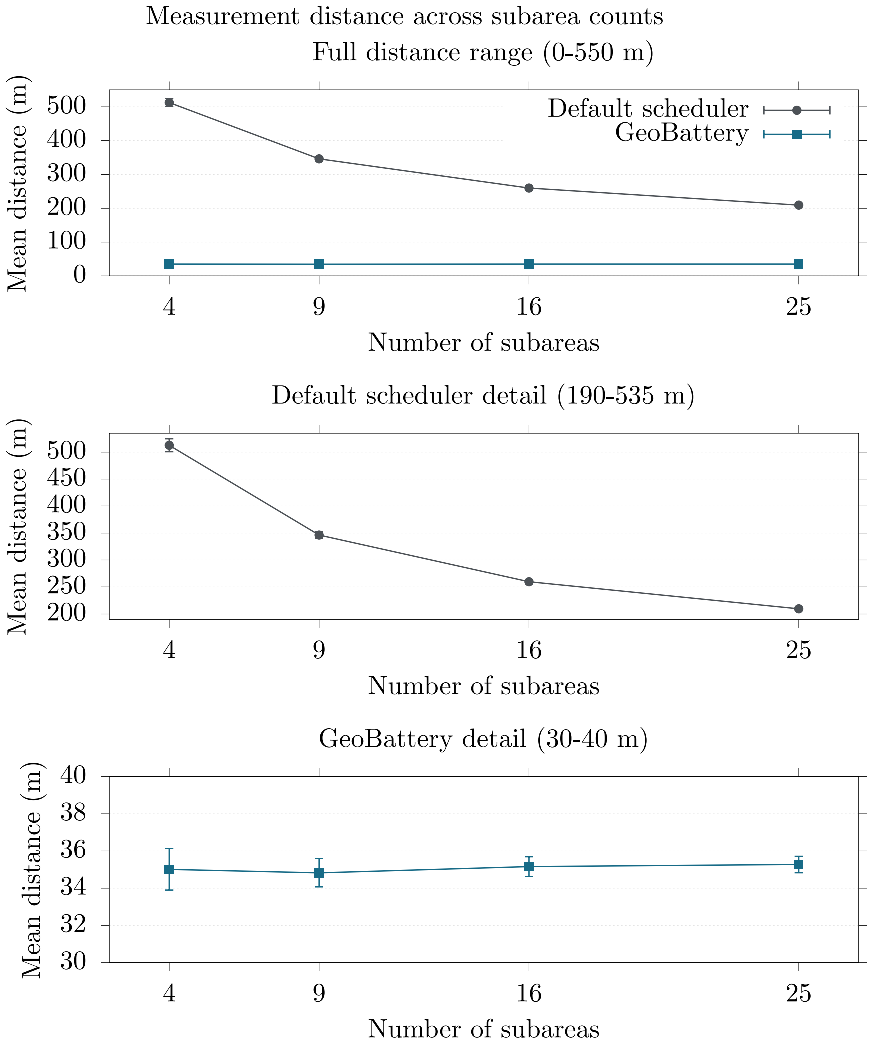

Figure S7: Mean distance from target to selected node across subarea counts. The top panel shows both schedulers on the full 0–550 m scale. The lower panels show the default scheduler (190–535 m) and GeoBattery (30–40 m) separately at a larger scale. Points are means of 30 run means. Error bars show 95% bootstrap confidence intervals. The y axis is labeled in each panel.
