# Supplementary material for: Geographic and Battery-Aware Kubernetes Scheduling for IoT Sensing
Source: Sensors (Basel). 2026 Nov 5;26(21):7003. doi: 10.3390/s26217003 (PMC13766508; doi:10.3390/s26217003)
Supplement: Supplementary file 1 [file sensors-26-07003-s001.zip › figure_S8.pdf]

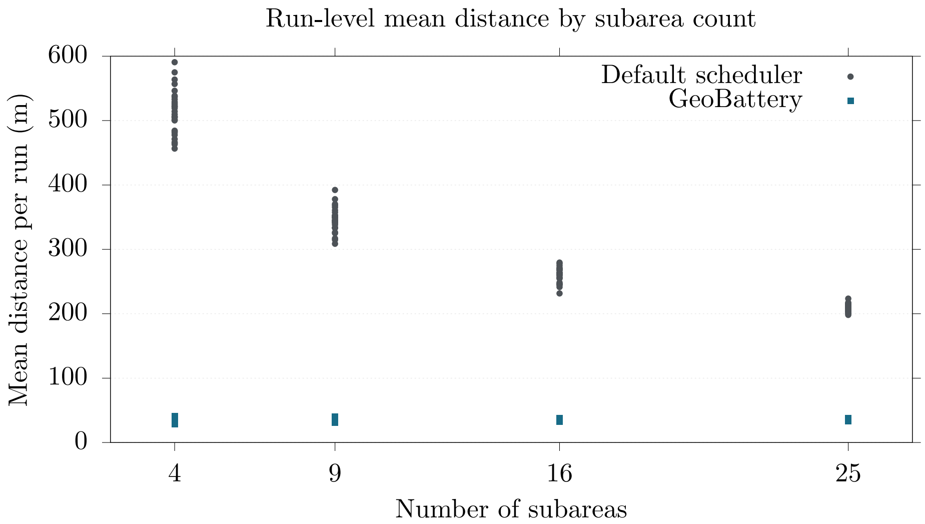

Figure S8: Mean distance from target to selected node in each of the 30 matched runs at every subarea count. Both schedulers share an x position for each count (4, 9, 16, or 25). Color distinguishes the default scheduler from GeoBattery. Each point represents one run.
