# Supplementary material for: Geographic and Battery-Aware Kubernetes Scheduling for IoT Sensing
Source: Sensors (Basel). 2026 Nov 5;26(21):7003. doi: 10.3390/s26217003 (PMC13766508; doi:10.3390/s26217003)
Supplement: Supplementary file 1 [file sensors-26-07003-s001.zip › figure_S9.pdf]

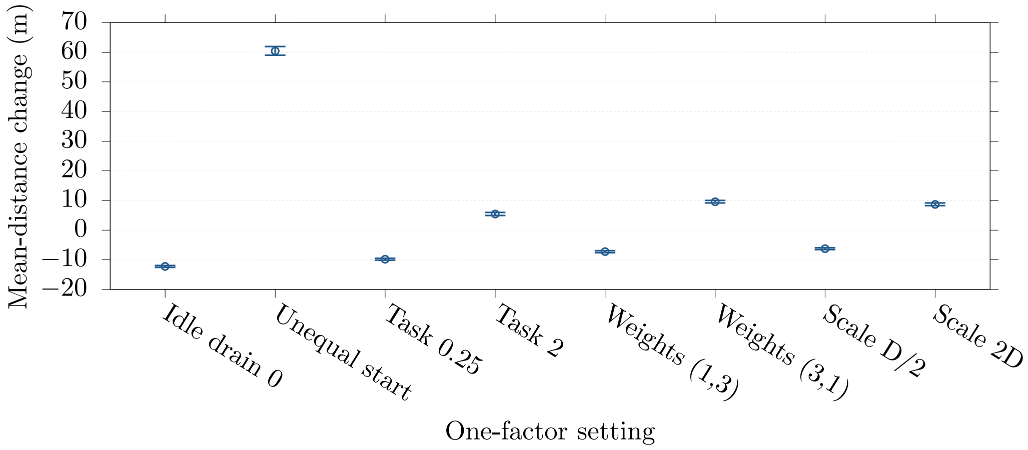

Figure S9: Paired change in mean distance from target to selected node for each single factor setting relative to the center combined policy. Points are mean differences across runs. Capped error bars are 95% paired percentile bootstrap intervals from 30 replicate IDs. Negative values denote shorter mean distance than the center setting.
