# Supplementary material for: Geographic and Battery-Aware Kubernetes Scheduling for IoT Sensing
Source: Sensors (Basel). 2026 Nov 5;26(21):7003. doi: 10.3390/s26217003 (PMC13766508; doi:10.3390/s26217003)
Supplement: Supplementary file 1 [file sensors-26-07003-s001.zip › figure_S10.pdf]

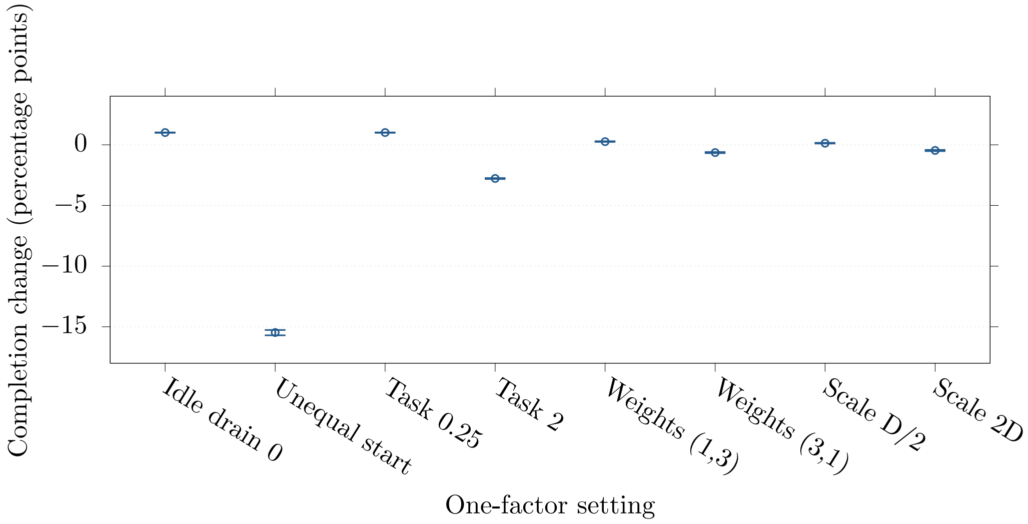


Figure S10: Paired change in request completion, in percentage points, for each single factor setting relative to the center combined policy. Points are mean differences across runs. Capped error bars are 95% paired percentile bootstrap intervals from 30 replicate IDs. Negative values denote lower completion than the center setting.
