# Supplementary material for: Geographic and Battery-Aware Kubernetes Scheduling for IoT Sensing
Source: Sensors (Basel). 2026 Nov 5;26(21):7003. doi: 10.3390/s26217003 (PMC13766508; doi:10.3390/s26217003)
Supplement: Supplementary file 1 [file sensors-26-07003-s001.zip › figure_S11.pdf]

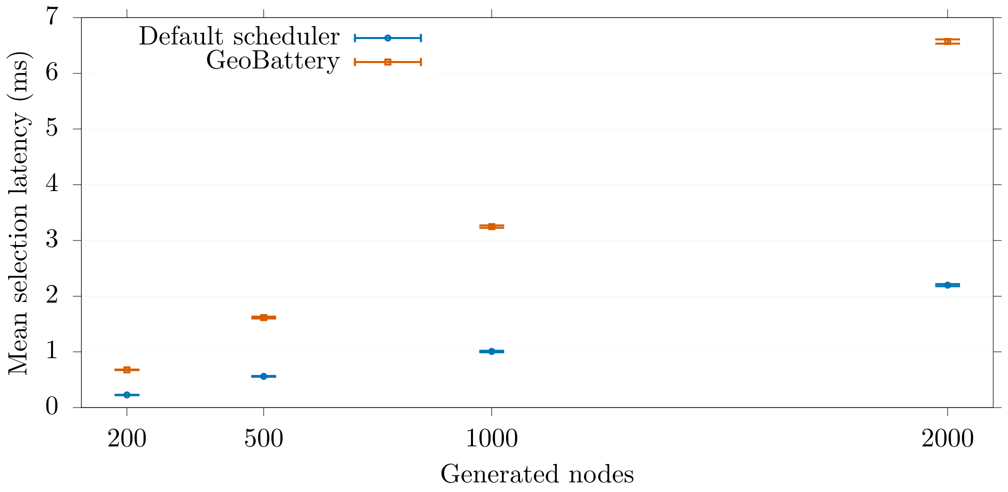

Figure S11: Mean selection latency across generated node counts. Points are means of 30 request means, one per run. Bars show 95% bootstrap confidence intervals across runs. The profiles use different candidate eligibility rules.
