# Supplementary material for: Geographic and Battery-Aware Kubernetes Scheduling for IoT Sensing
Source: Sensors (Basel). 2026 Nov 5;26(21):7003. doi: 10.3390/s26217003 (PMC13766508; doi:10.3390/s26217003)
Supplement: Supplementary file 1 [file sensors-26-07003-s001.zip › figure_S13.pdf]

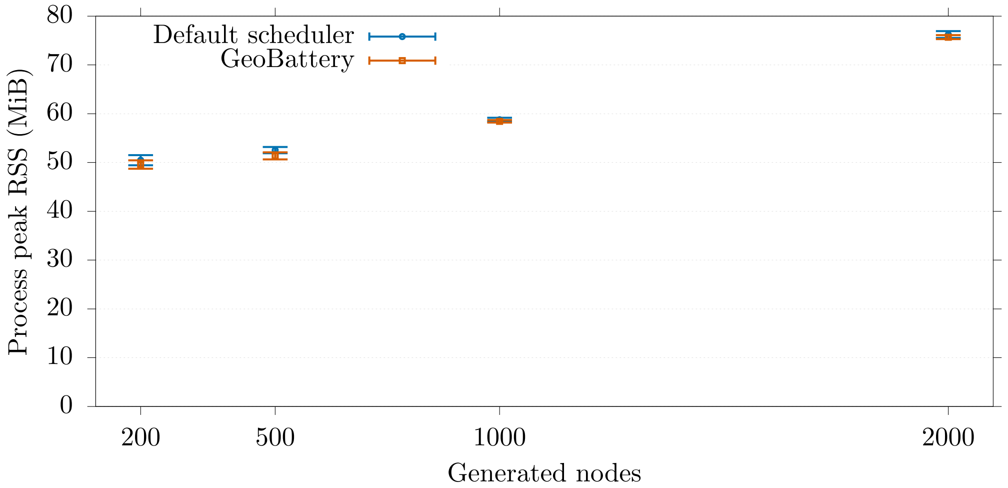

Figure S13: Process peak resident set size sampled at simulation completion, in MiB. Points are 30-run means with 95% run-bootstrap confidence intervals.
