# Supplementary material for: Geographic and Battery-Aware Kubernetes Scheduling for IoT Sensing
Source: Sensors (Basel). 2026 Nov 5;26(21):7003. doi: 10.3390/s26217003 (PMC13766508; doi:10.3390/s26217003)
Supplement: Supplementary file 1 [file sensors-26-07003-s001.zip › table_S1.pdf]

Table S1: GeoBattery's runtime interface and validation roles.

Data	Kubernetes Field	Validation or Use
Target x, y	Pod annotations `iot.geobattery/x-m`, `iot.geobattery/y-m`	Finite values in meters
Node x, y	Node labels `iot.example.org/x-m`, `iot.example.org/y-m`	Finite values in the shared frame
Battery level	Node conditions `BATTERYLEVEL1`–`BATTERYLEVEL10`	Unique True level, remaining present levels False
Heartbeat	Active battery condition	Age in $[0, \tau]$ at cycle time
Weights and scale	Plugin configuration	Finite weights, positive sum, positive scale
