# Supplementary material for: Geographic and Battery-Aware Kubernetes Scheduling for IoT Sensing
Source: Sensors (Basel). 2026 Nov 5;26(21):7003. doi: 10.3390/s26217003 (PMC13766508; doi:10.3390/s26217003)
Supplement: Supplementary file 1 [file sensors-26-07003-s001.zip › table_S2.pdf]

Table S2: GeoBattery request, node, and policy inputs.

| Input | Location | Role in the Model |
|---|---|---|
| Target coordinates $(x_p, y_p)$ | `iot.geobattery/x-m`, `iot.geobattery/y-m` | Define the requested point in local meters |
| Node coordinates $(x_i, y_i)$ | `iot.example.org/x-m`, `iot.example.org/y-m` | Define the worker location in the same local frame |
| Battery level and heartbeat | Node conditions | Provide a fresh ordinal state used for validation and scoring |
| Battery/proximity weights | Scheduler configuration | Set the relative contribution of each preference |
| Proximity scale $D$ | Scheduler configuration | Normalize distance into a score contribution |
| Maximum battery age $\tau$ | Scheduler configuration | Bound the acceptable age of the active heartbeat |
