# Supplementary material for: Geographic and Battery-Aware Kubernetes Scheduling for IoT Sensing
Source: Sensors (Basel). 2026 Nov 5;26(21):7003. doi: 10.3390/s26217003 (PMC13766508; doi:10.3390/s26217003)
Supplement: Supplementary file 1 [file sensors-26-07003-s001.zip › table_S3.pdf]

Table S3: Mean distance from target to node and paired differences between the default scheduler and GeoBattery across subarea counts. Values are in meters. Intervals are 95% bootstrap confidence intervals across 30 matched seeds.

**Subareas**	**Default Scheduler Mean (95% CI)**	**GeoBattery Mean (95% CI)**	**Paired Distance Difference (95% CI)**
4	512.46 (500.66–524.68)	35.01 (33.90–36.13)	477.44 (465.44–489.38)
9	346.08 (339.69–352.68)	34.83 (34.07–35.60)	311.26 (304.91–317.80)
16	259.79 (255.54–263.81)	35.16 (34.63–35.69)	224.63 (220.41–228.64)
25	209.44 (207.48–211.44)	35.28 (34.83–35.72)	174.16 (172.26–176.06)
