# Supplementary material for: Geographic and Battery-Aware Kubernetes Scheduling for IoT Sensing
Source: Sensors (Basel). 2026 Nov 5;26(21):7003. doi: 10.3390/s26217003 (PMC13766508; doi:10.3390/s26217003)
Supplement: Supplementary file 1 [file sensors-26-07003-s001.zip › table_S4.pdf]

Table S4: Mean distance and request completion in the center condition across 30 runs. Each entry gives the mean and 95% percentile bootstrap interval across runs.

**Policy**	**Mean Distance (m) [95% CI]**	**Completion (%) [95% CI]**
Proximity-only $(0, 1)$	37.437 [37.177, 37.690]	99.9675 [99.9358, 99.9917]
Battery-only $(1, 0)$	1042.850 [1039.073, 1046.716]	98.0000 [98.0000, 98.0000]
Combined $(1, 1)$	47.868 [47.523, 48.227]	98.9958 [98.9767, 99.0117]
Default-profile reference	521.509 [520.047, 522.909]	99.1867 [99.1592, 99.2158]
