# Supplementary material for: Geographic and Battery-Aware Kubernetes Scheduling for IoT Sensing
Source: Sensors (Basel). 2026 Nov 5;26(21):7003. doi: 10.3390/s26217003 (PMC13766508; doi:10.3390/s26217003)
Supplement: Supplementary file 1 [file sensors-26-07003-s001.zip › table_S5.pdf]

Table S5: Restricted mean modeled lifetime, median node death time, and survival after timestep 1000 in the center condition. Restricted mean lifetime values are means across 30 runs with 95% percentile bootstrap intervals across runs. Median node death times are descriptive medians across the 30,000 observed node death times for each policy.

**Policy**	**Restricted Mean Lifetime (Steps) [95% CI]**	**Median Node Death Time (Steps)**	**Survival After Timestep 1000 (%)**
Proximity-only $(0, 1)$	960.757 [960.740, 960.773]	969	0
Battery-only $(1, 0)$	961.296 [961.296, 961.296]	960	0
Combined $(1, 1)$	961.135 [961.118, 961.152]	960	0
Default-profile reference	960.890 [960.874, 960.905]	960	0
