# Supplementary material for: Geographic and Battery-Aware Kubernetes Scheduling for IoT Sensing
Source: Sensors (Basel). 2026 Nov 5;26(21):7003. doi: 10.3390/s26217003 (PMC13766508; doi:10.3390/s26217003)
Supplement: Supplementary file 1 [file sensors-26-07003-s001.zip › table_S6.pdf]

Table S6: Distance and completion effects for combined scoring relative to each matched score ablation in the center condition. Values are mean paired differences with 95% paired percentile bootstrap intervals. Neither contrast changed the proportion surviving after timestep 1000.

**Paired Contrast**	**Distance Difference (m) [95% CI]**	**Completion Difference (Percentage Points) [95% CI]**
Combined − proximity-only	+10.430 [+10.065, +10.804]	−0.972 [−1.000, −0.939]
Combined − battery-only	−994.983 [−998.907, −991.238]	+0.996 [+0.977, +1.012]
