# Supplementary material for: Geographic and Battery-Aware Kubernetes Scheduling for IoT Sensing
Source: Sensors (Basel). 2026 Nov 5;26(21):7003. doi: 10.3390/s26217003 (PMC13766508; doi:10.3390/s26217003)
Supplement: Supplementary file 1 [file sensors-26-07003-s001.zip › table_S7.pdf]

Table S7: One-factor changes in restricted mean modeled lifetime and the proportion of nodes surviving through timestep 1000 relative to the center combined-policy condition. Values are mean paired differences with 95% paired percentile-bootstrap intervals across 30 run IDs.

**One-Factor Setting**	**Restricted Mean Lifetime Difference (Steps)**	**Surviving Nodes After Timestep 1000 Difference (Percentage Points)**
Zero idle drain	+38.865 [+38.848, +38.881]	+100 [+100, +100]
Unequal initial battery	−444.113 [−447.060, −441.117]	0 [0, 0]
Task drain 0.25	+29.179 [+29.161, +29.196]	0 [0, 0]
Task drain 2	−35.991 [−36.025, −35.956]	0 [0, 0]
Weights $(1, 3)$	−0.149 [−0.170, −0.128]	0 [0, 0]
Weights $(3, 1)$	+0.099 [+0.077, +0.119]	0 [0, 0]
Distance scale $D/2$	−0.079 [−0.097, −0.061]	0 [0, 0]
Distance scale $2D$	+0.073 [+0.051, +0.095]	0 [0, 0]
