# Supplementary material for: Geographic and Battery-Aware Kubernetes Scheduling for IoT Sensing
Source: Sensors (Basel). 2026 Nov 5;26(21):7003. doi: 10.3390/s26217003 (PMC13766508; doi:10.3390/s26217003)
Supplement: Supplementary file 1 [file sensors-26-07003-s001.zip › supplementary_material.pdf]

Supplementary Material

Geographic and Battery-Aware Kubernetes Scheduling for IoT Sensing

George Koufoudakis

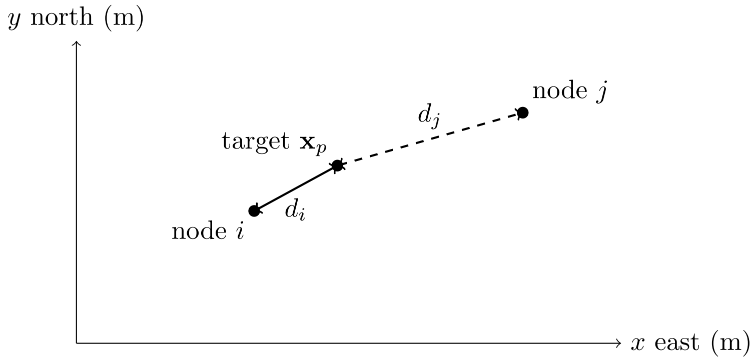

Figure S1: Local Cartesian representation of the target point and two worker nodes. The solid line shows the distance from the target to node i, and the dashed line shows the distance from the target to node j. Sensing-coverage regions and maximum-range boundaries are separate application constraints.

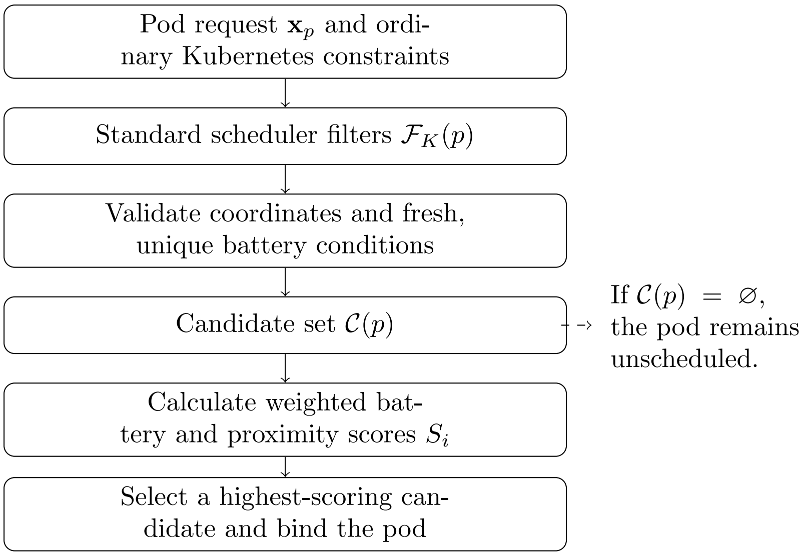

Figure S2: GeoBattery scheduling model from pod request to node selection. Solid arrows show the scheduling flow; the dashed arrow points to the note that the pod remains unscheduled if there are no candidates. The custom policy validates and scores candidates within the standard Kubernetes scheduling cycle.

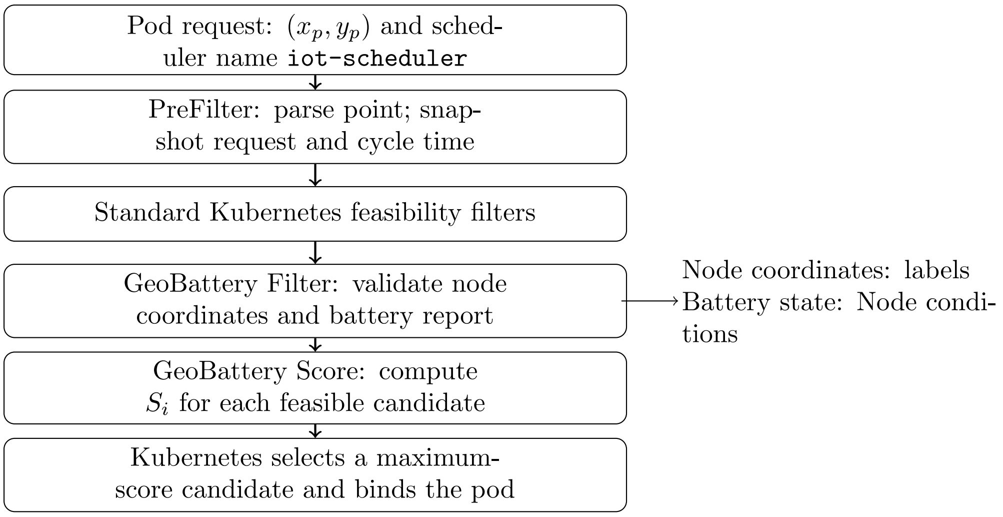

Figure S3: GeoBattery's Scheduling Framework path. Standard filters and plugin validation establish the feasible set before score-based node selection.

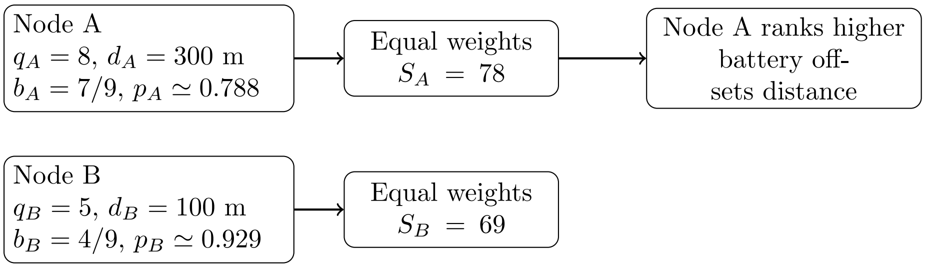

Figure S4: Illustrative score trade-off under equal battery and proximity weights. Node B is closer, while Node A's higher battery band produces the larger rounded score. Values are calculated from the stated score model.

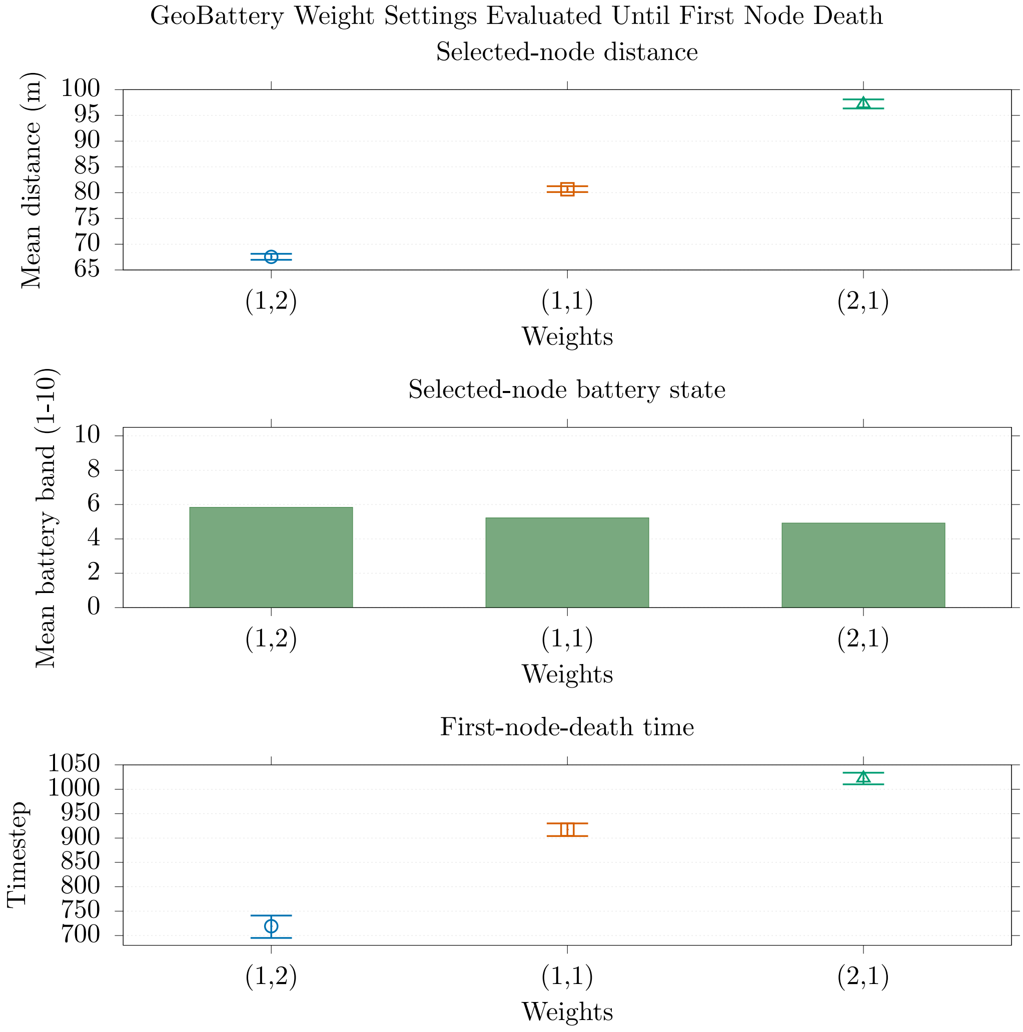

Figure S5: GeoBattery weight settings evaluated until the first node death. Each setting was tested over 30 runs with matched seeds. Symbols distinguish the settings. Each weight pair (w_b, w_p) gives the battery and proximity weights. Lower distance, a higher battery category, and later first death represent the respective objectives.

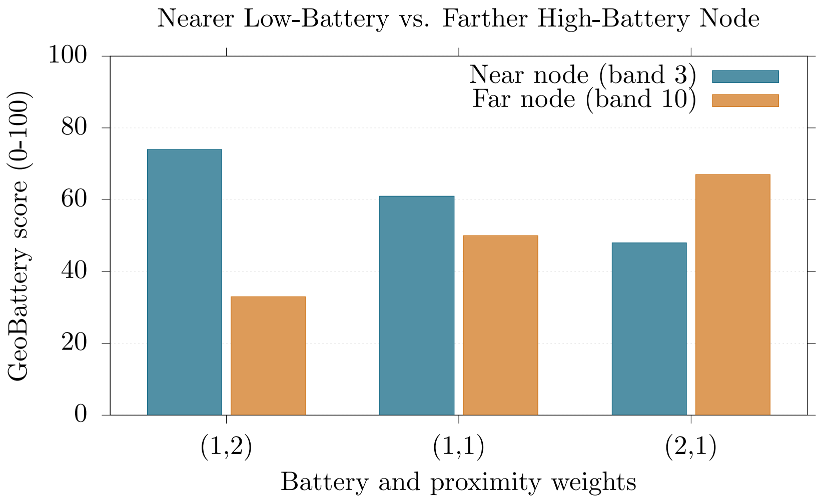

Figure S6: GeoBattery scores in a controlled comparison between a nearer, lower-battery node and a farther, higher-battery node. The scheduler selects the higher-scoring candidate: the near node for $(1, 2)$ and $(1, 1)$, and the far node for $(2, 1)$. Weight pairs are ordered as battery weight followed by proximity weight.

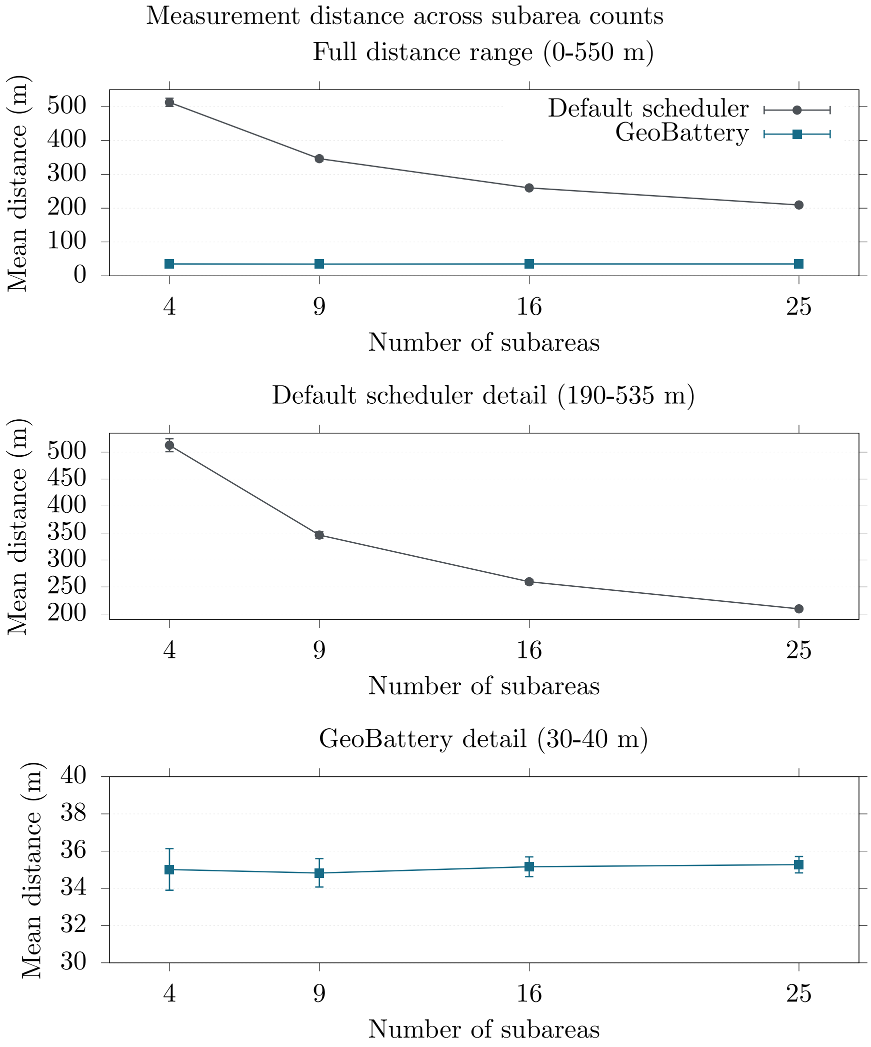

Figure S7: Mean distance from target to selected node across subarea counts. The top panel shows both schedulers on the full 0–550 m scale. The lower panels show the default scheduler (190–535 m) and GeoBattery (30–40 m) separately at a larger scale. Points are means of 30 run means. Error bars show 95% bootstrap confidence intervals. The y axis is labeled in each panel.

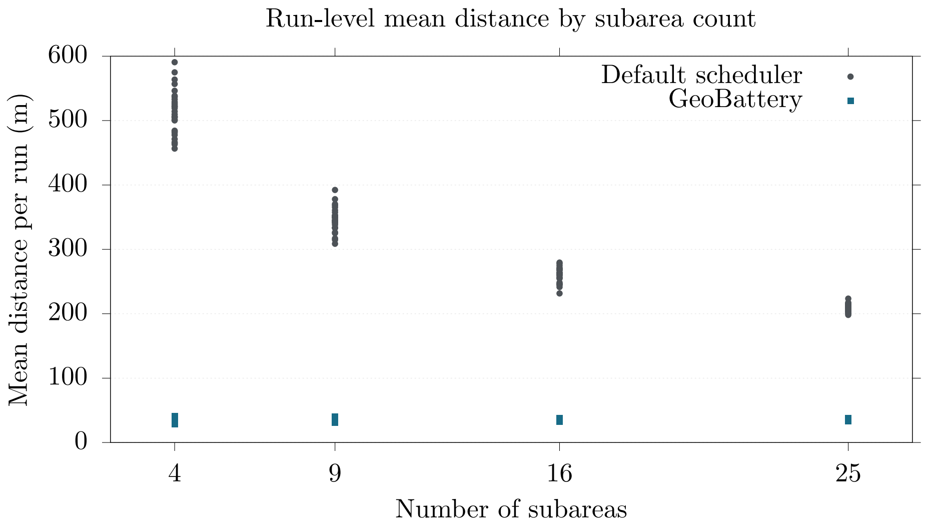

Figure S8: Mean distance from target to selected node in each of the 30 matched runs at every subarea count. Both schedulers share an x position for each count (4, 9, 16, or 25). Color distinguishes the default scheduler from GeoBattery. Each point represents one run.

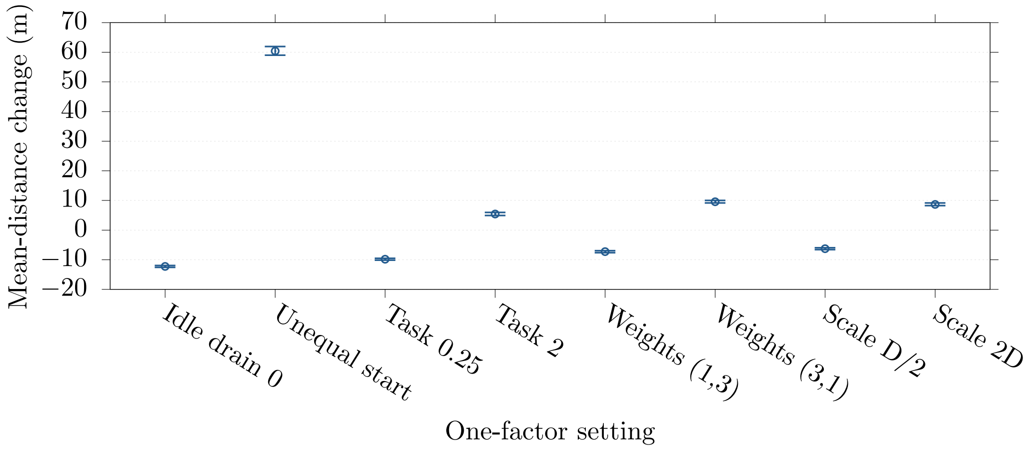

Figure S9: Paired change in mean distance from target to selected node for each single factor setting relative to the center combined policy. Points are mean differences across runs. Capped error bars are 95% paired percentile bootstrap intervals from 30 replicate IDs. Negative values denote shorter mean distance than the center setting.

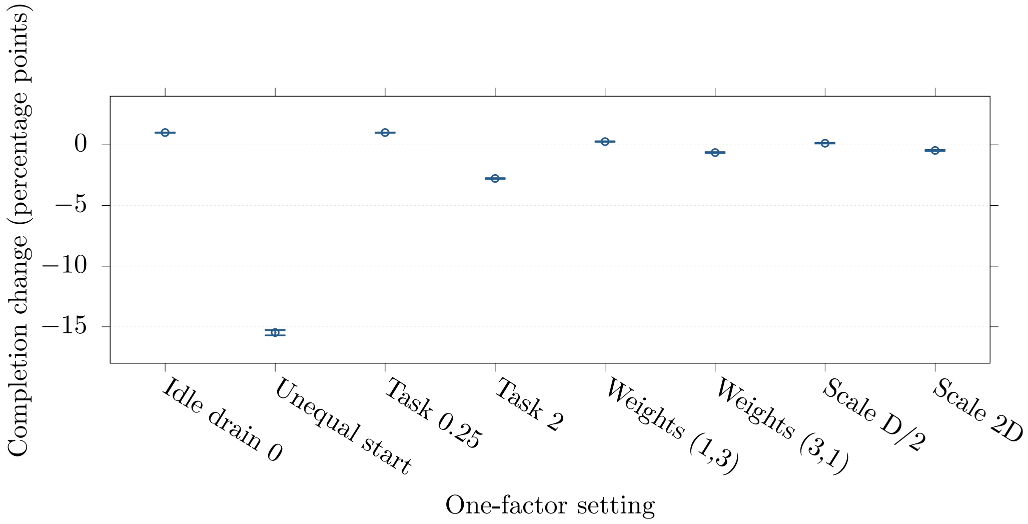

Figure S10: Paired change in request completion, in percentage points, for each single factor setting relative to the center combined policy. Points are mean differences across runs. Capped error bars are 95% paired percentile bootstrap intervals from 30 replicate IDs. Negative values denote lower completion than the center setting.

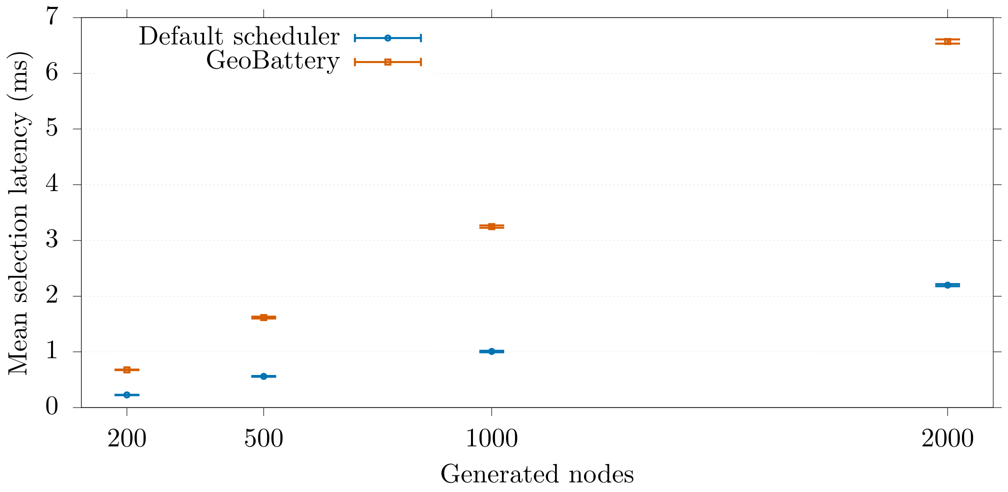

Figure S11: Mean selection latency across generated node counts. Points are means of 30 request means, one per run. Bars show 95% bootstrap confidence intervals across runs. The profiles use different candidate eligibility rules.

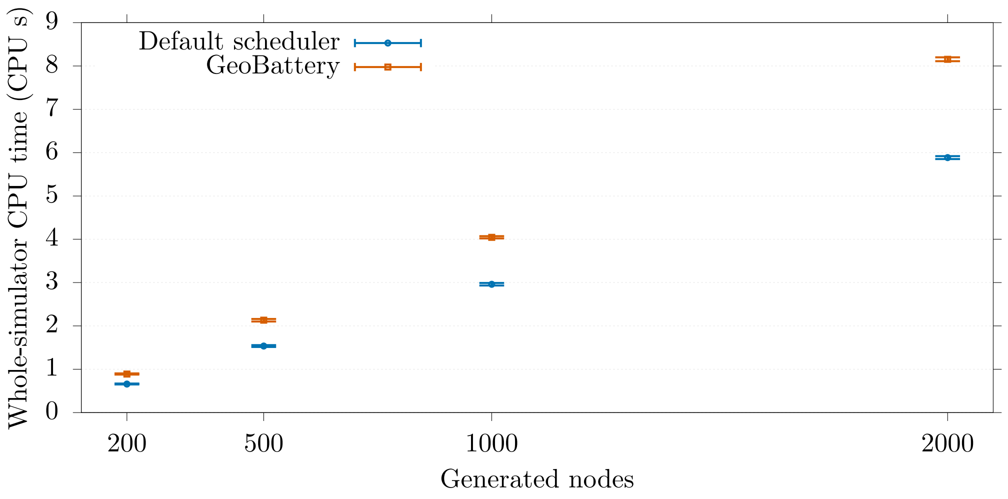

Figure S12: Whole-simulator CPU time per process, including initialization, requests, and output. User and system CPU time are summed over all threads. Points are 30-run means with 95% run-bootstrap confidence intervals.

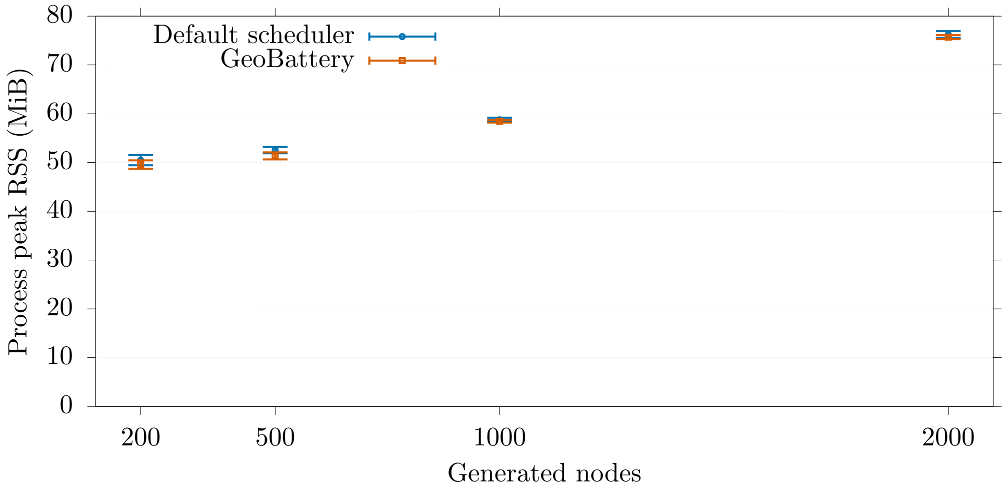

Figure S13: Process peak resident set size sampled at simulation completion, in MiB. Points are 30-run means with 95% run-bootstrap confidence intervals.

Table S1: GeoBattery's runtime interface and validation roles.

Data	Kubernetes Field	Validation or Use
Target x, y	Pod annotations `iot.geobattery/x-m`, `iot.geobattery/y-m`	Finite values in meters
Node x, y	Node labels `iot.example.org/x-m`, `iot.example.org/y-m`	Finite values in the shared frame
Battery level	Node conditions `BATTERYLEVEL1`–`BATTERYLEVEL10`	Unique True level, remaining present levels False
Heartbeat	Active battery condition	Age in $[0, \tau]$ at cycle time
Weights and scale	Plugin configuration	Finite weights, positive sum, positive scale

Table S2: GeoBattery request, node, and policy inputs.

Input	Location	Role in the Model
Target coordinates (x_p, y_p)	`iot.geobattery/x-m`, `iot.geobattery/y-m`	Define the requested point in local meters
Node coordinates (x_i, y_i)	`iot.example.org/x-m`, `iot.example.org/y-m`	Define the worker location in the same local frame
Battery level and heartbeat	Node conditions	Provide a fresh ordinal state used for validation and scoring
Battery/proximity weights	Scheduler configuration	Set the relative contribution of each preference
Proximity scale D	Scheduler configuration	Normalize distance into a score contribution
Maximum battery age τ	Scheduler configuration	Bound the acceptable age of the active heartbeat

Table S3: Mean distance from target to node and paired differences between the default scheduler and GeoBattery across subarea counts. Values are in meters. Intervals are 95% bootstrap confidence intervals across 30 matched seeds.

**Subareas**	**Default Scheduler Mean (95% CI)**	**GeoBattery Mean (95% CI)**	**Paired Distance Difference (95% CI)**
4	512.46 (500.66–524.68)	35.01 (33.90–36.13)	477.44 (465.44–489.38)
9	346.08 (339.69–352.68)	34.83 (34.07–35.60)	311.26 (304.91–317.80)
16	259.79 (255.54–263.81)	35.16 (34.63–35.69)	224.63 (220.41–228.64)
25	209.44 (207.48–211.44)	35.28 (34.83–35.72)	174.16 (172.26–176.06)

Table S4: Mean distance and request completion in the center condition across 30 runs. Each entry gives the mean and 95% percentile bootstrap interval across runs.

**Policy**	**Mean Distance (m) [95% CI]**	**Completion (%) [95% CI]**
Proximity-only $(0, 1)$	37.437 [37.177, 37.690]	99.9675 [99.9358, 99.9917]
Battery-only $(1, 0)$	1042.850 [1039.073, 1046.716]	98.0000 [98.0000, 98.0000]
Combined $(1, 1)$	47.868 [47.523, 48.227]	98.9958 [98.9767, 99.0117]
Default-profile reference	521.509 [520.047, 522.909]	99.1867 [99.1592, 99.2158]

Table S5: Restricted mean modeled lifetime, median node death time, and survival after timestep 1000 in the center condition. Restricted mean lifetime values are means across 30 runs with 95% percentile bootstrap intervals across runs. Median node death times are descriptive medians across the 30,000 observed node death times for each policy.

**Policy**	**Restricted Mean Lifetime (Steps) [95% CI]**	**Median Node Death Time (Steps)**	**Survival After Timestep 1000 (%)**
Proximity-only $(0, 1)$	960.757 [960.740, 960.773]	969	0
Battery-only $(1, 0)$	961.296 [961.296, 961.296]	960	0
Combined $(1, 1)$	961.135 [961.118, 961.152]	960	0
Default-profile reference	960.890 [960.874, 960.905]	960	0

Table S6: Distance and completion effects for combined scoring relative to each matched score ablation in the center condition. Values are mean paired differences with 95% paired percentile bootstrap intervals. Neither contrast changed the proportion surviving after timestep 1000.

**Paired Contrast**	**Distance Difference (m) [95% CI]**	**Completion Difference (Percentage Points) [95% CI]**
Combined − proximity-only	+10.430 [+10.065, +10.804]	−0.972 [−1.000, −0.939]
Combined − battery-only	−994.983 [−998.907, −991.238]	+0.996 [+0.977, +1.012]

Table S7: One-factor changes in restricted mean modeled lifetime and the proportion of nodes surviving through timestep 1000 relative to the center combined-policy condition. Values are mean paired differences with 95% paired percentile-bootstrap intervals across 30 run IDs.

**One-Factor Setting**	**Restricted Mean Lifetime Difference (Steps)**	**Surviving Nodes After Timestep 1000 Difference (Percentage Points)**
Zero idle drain	+38.865 [+38.848, +38.881]	+100 [+100, +100]
Unequal initial battery	−444.113 [−447.060, −441.117]	0 [0, 0]
Task drain 0.25	+29.179 [+29.161, +29.196]	0 [0, 0]
Task drain 2	−35.991 [−36.025, −35.956]	0 [0, 0]
Weights $(1, 3)$	−0.149 [−0.170, −0.128]	0 [0, 0]
Weights $(3, 1)$	+0.099 [+0.077, +0.119]	0 [0, 0]
Distance scale $D/2$	−0.079 [−0.097, −0.061]	0 [0, 0]
Distance scale $2D$	+0.073 [+0.051, +0.095]	0 [0, 0]
